# Barley Stem Bending Resistance Declines During Maturation, Then Peaks in Ripe, Dry Plants

**DOI:** 10.3390/plants15081234

**Published:** 2026-04-17

**Authors:** Alberto Gianinetti, Marina Baronchelli

**Affiliations:** Research Centre for Genomics and Bioinformatics, Council for Agricultural Research and Economics (CREA), Via S. Protaso 302, 29017 Fiorenzuola d’Arda, Italy; marina.baronchelli@crea.gov.it

**Keywords:** barley lodging, bending resistance, culm strength, pulvinus, strength dynamic changes

## Abstract

Barley lodging—specifically stem lodging—occurs when the bending moments from wind and ear weight exceed the culm’s load-bearing capacity. Lodging risk decreases as plant height decreases and culm strength increases. Geometry (stem diameter, culm wall thickness) and material strength determine culm bending strength. By studying changes in stem mechanical properties (at three positions along the culm) in two genotypes (grown in a greenhouse), we found that culm strength (assessed with a three-point bending test) slightly diminished through ripening owing to a decline in both area moment of inertia (i.e., strength due to geometry alone) and apparent material strength, presumably due to turgor loss. When the stem segments collected from fully ripe plants were dried to a moisture content typical of harvest maturity, however, strength rose to a maximum. Thus, minimum stem bending resistance occurs during a window in which plants are fully ripe but have not yet reached harvest-dry moisture content. Hence, in the absence of rain—which would severely reduce the mechanical strength of dry, ripe plants—the physiological risk of stem lodging is highest when the crop is fully ripe but not yet harvest-dry. However, the actual lodging risk increases as harvest approaches, because summer storms are frequent at this time of year and dry straw loses rigidity when wetted.

## 1. Introduction

Lodging is the permanent bending of crop stems away from their upright position—sometimes even down to near ground level—caused by wind and rain [1,2]. Barley lodging can occur because of anchorage failure (root lodging), buckling of the stem base (stem lodging) or buckling of the upper half of the stem (brackling) [2]. In general, barley is more prone to stem buckling than root lodging [3], with the former being more likely during the late cycle season, whereas the latter is more common earlier.

Lodging resistance depends on the mechanical properties of stems. Relevant parameters [2] are described below.

The area moment of inertia, *I*, is the geometrical component of the resistance to bending that depends solely on how the material of the cross-section of the object is distributed relative to the bending axis: the more an object has material located farther from the bending axis the stiffer and stronger it is along that axis. This is why the culms of several grasses are hollow cylinders [4,5]. *I* is calculated as [6]

(1) *I* = π/4 × (*r*_e_^4^ − *r*_i_^4^),

where *r*_e_ is the outside radius of the culm, and *r*_i_ is the inside radius of the culm (that is, *r*_e_ minus the width of the culm wall). Stiff stems with large diameter and thick culm wall better resist buckling, whereas tall, slender stems with low rigidity are more failure-prone [1].

Wind produces on the stalk a bending moment that is opposed by a bending–resisting moment dependent on the mechanical properties of the stem [1,7]. Beyond the maximum bending–resisting moment the culm can withstand, which is also known as the failure moment, or stem bending strength (*B_S_*), structural failure occurs, either by breaking or creeping [2]. When the bending moment exceeds *B_S_*, therefore, bending cannot be reversed, and plants lodge [2]. Besides plant height, therefore, *B_S_* is a key mechanical determinant of lodging resistance. It depends on the shape of the cross-section, that is, *I*, and the material strength (σ_b_), which is the maximum stress that a material can withstand before failing [2,5]:

(2) *B_S_* = σ_b_ × *I*/*r*_e_

In green plants, turgid cells maintain rigidity because hydrostatic pressure (turgor)—exerted by the intracellular water solution—presses the plasma membrane against the cell wall, which in turn resists outward pressure and keeps the tissue firm [8]. Thus, in non-lignified, soft herbaceous plants, turgor pressure supports compressive loading, while primary cell walls bear tensile stress [8]. In taller herbaceous stems, thickened cell walls of collenchymatous and sclerenchymatous tissues are the principal anatomical structures supporting plants [5]. These tissues are dominated by cellulose, hemicellulose, and lignin (in sclerenchyma), which are therefore fundamentally linked to stem lodging resistance [9].

Many of these taller herbaceous species, like barley, have hollow stems to reduce weight and material costs while retaining sufficient rigidity for support [5]. These plants, therefore, show a ‘hydro-skeleton’ in which a sclerenchymatous rind enhances bending resistance, as secondary cell walls have greater resistance to both compressive and tensile stresses [5,8]. Mechanical properties of plants, indeed, depend on cell wall constituents [10]. Thus, whereas in soft herbaceous plants loss of water and turgor pressure makes tissues limp, causing wilting or drooping, in cereals, stems remain upright because their rigidity is largely maintained by partially lignified structural tissues even when turgor declines [1,11,12,13].

In barley, like in other small grain cereals, the base of each internode just above the node—which is the point where transversal septa of adjacent internodes juxtapose—remains meristematic and soft, lacking lignification until after heading, to allow secondary growth during gravitropic response to lodging [14]. This short portion of the internode is, therefore, a weak zone in the stem [15]. However, in green barley plants, the leaf sheath (which originates at the node) is swollen to form the meristematic pulvinus, a hard knot immediately above the node that braces and surrounds the weak—not swollen—base of the internode, reinforcing it [5,15]. As pulvini provide a strong gravitropic response, their cell walls have poor secondary modifications, and their sturdiness is, therefore, chiefly due to turgor pressure [5], so that they shrink as they dry out [16].

When the plant is green, stem buckling practically never takes place at the pulvinus. Rather, it occurs along the internode, at least half a centimeter below the node/pulvinus complex, but rarely above it, because the presence of the leaf sheath, which tightly envelops the stem and thus strengthens it, makes this occurrence less probable [16]. When the plant is fully senescent, instead, stem failure most often takes place at the pulvinus, which is the mechanical weak point for bending resistance in dead-ripe plants [16]. There is, therefore, a change in the mechanical properties of the culm—in particular of the pulvini—during the passage from green, turgid plants to senescent ones. The literature on this transition is extremely scant, however.

Since stem lodging is most common during the late stages of crop development—generally in the final weeks before harvest, when plants have senesced and are drying down [1,7]—the present work aims to delineate the timeframe of the changes in the main mechanical properties of barley through ripening. Two widely different genotypes were used in this study: cultivar Ketos and landrace Tibet-A4. The former is lodging-resistant whereas the latter is highly susceptible to lodging [16]. *B_S_* was assessed on stem segments collected at three positions along the stem—bottom, middle, and upper—by manually (i.e., with a fingertip) loading stem segments in a three-point bending test.

## 2. Results

The overall design of our experiment involved growing two barley genotypes (Ketos and Tibet-A4) in a fully randomized greenhouse setup, with 16 pots per genotype as independent experimental units and 3–4 plants per pot serving as the biological replicates. Stem sampling was performed from early dough (late April) through harvest maturity (mid-June) at multiple dates for each genotype, aiming to collect material through subsequent BBCH (Biologische Bundesanstalt, Bundessortenamt und CHemische Industrie [17]) developmental stages. However, because the two genotypes differ in phenology (Tibet-A4 flowering earlier and Ketos later), one sampling stage ultimately did not end up temporally aligned between genotypes. At each sampling event, stem material was collected from the available plants, and the largest and structurally dominant culms were measured, yielding approximately 15 culms per genotype per sampling time. From each culm, three defined stem segments (basal, middle, and upper) were excised and measured for biometric traits and bending properties. This design ensured biological replication at the plant/culm level and complete randomization of the experimental units at establishment.

**Figure 1:**
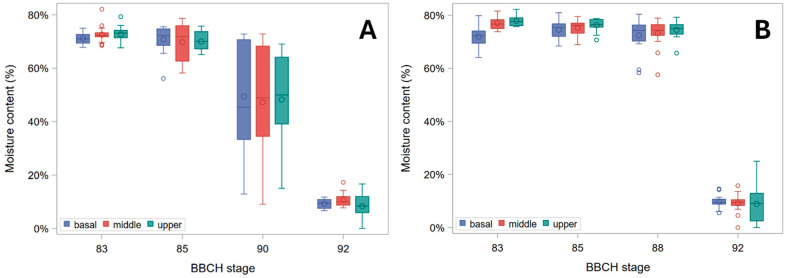
Water content of stem segments. Box plots for the basal, middle, and upper stem segments at subsequent phenological development stages (BBCH scale) are shown. (**A**) Cultivar Ketos. (**B**) Landrace Tibet-A4. Stages: 83 (early dough), 85 (soft dough), 88 (all grains solid, about half hard), 90 (grain hard, drying-down), 92 (harvest ripe). Moisture assessment (by oven drying) was carried out immediately after collecting at all stages but the last, for which water content was measured after further drying at RT for over five months. Each box shows the interquartile range (IQR), from the 25th percentile (Q1) to the 75th percentile (Q3). Inside each box, the short line represents the median, and the circle is the mean value. The whiskers extend to the smallest and largest values within 1.5 × IQR of the box. Points beyond the whiskers are plotted as outliers.

Figure 1 shows that the water content of barley stem segments was relatively constant at 65–75% across BBCH stages 83 (early dough) to 85 (soft dough) for Ketos, and at 70–80% across BBCH stages 83 to 88 (all grains solid, about half hard) for Tibet-A4. As sampling stage differed between genotypes, results are shown separately for each genotype. At BBCH stage 90 (grain hard, drying-down), Ketos showed a drop in water content with very high variability among stems (Figure 1). Both genotypes displayed an average moisture content of around 10% after they were collected at BBCH stage 92 (harvest ripe) and further dried at room temperature (RT) for over five months to approximate field harvest-dry conditions. As typically observed, yellowing of leaves and stems started from the lower leaves at about BBCH stage 83 and progressed upwards, with the flag leaf usually fully senesced by BBCH stage 87. Up to physiological maturity (BBCH stages 88–89), therefore, leaf and culm yellowing was not associated with an apparent loss of water content. In other words, the observed stability of stem water content up to physiological maturity indicates that the initial senescence-related yellowing of leaves and culms does not immediately translate into stem desiccation.

**Figure 2:**
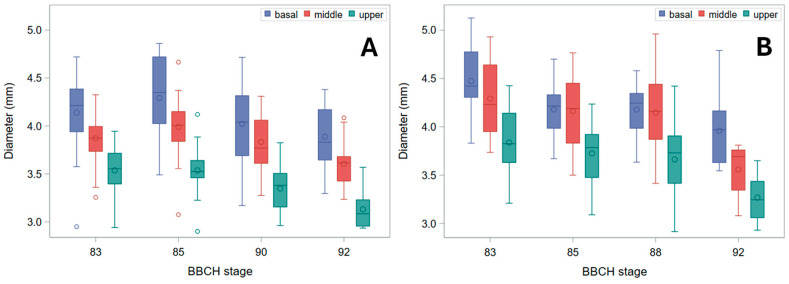
Diameter at node. Box plots for the basal, middle, and upper stem segments at subsequent phenological development stages (BBCH scale) are shown. (**A**) Cultivar Ketos. (**B**) Landrace Tibet-A4. Measurements were carried out immediately after collecting at all stages but the last, for which diameter was measured after further drying at RT for over five months.

Both genotypes had quite large stems, with diameters decreasing acropetally as well as through ripening (Figure 2).

**Figure 3:**
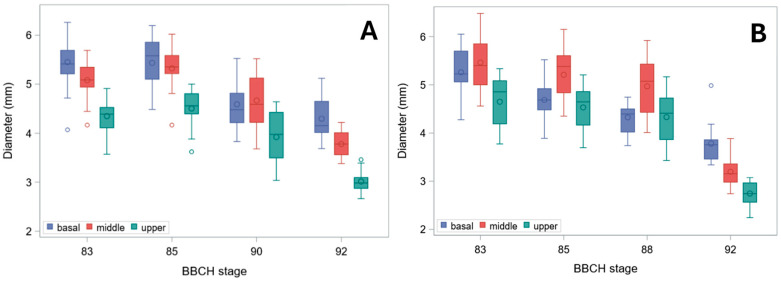
Diameter at pulvinus. Box plots for the basal, middle, and upper stem segments at subsequent phenological development stages (BBCH scale) are shown. (**A**) Cultivar Ketos. (**B**) Landrace Tibet-A4. Measurements were carried out immediately after collecting at all stages but the last, for which diameter was measured after further drying at RT for over five months.

At BBCH stage 92 (harvest ripe), the reduction in pulvinus diameter (Figure 3) was stronger than that of the node, especially for landrace Tibet-A4 (compare with Figure 2).

**Figure 4:**
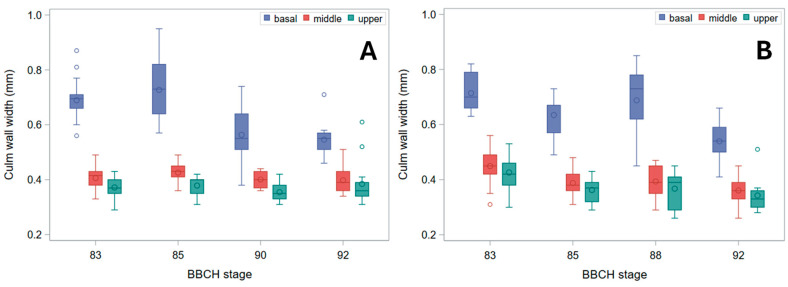
Culm wall thickness (*r*_e_ − *r*_i_) just below the node. Box plots for the basal, middle, and upper stem segments at subsequent phenological development stages (BBCH scale) are shown. (**A**) Cultivar Ketos. (**B**) Landrace Tibet-A4. Measurements were carried out immediately after collecting at all stages but the last, for which *r*_e_ − *r*_i_ was measured after further drying at RT for over five months.

A decrease in stem wall thickness from the base to the top of the culm was also observed, with a sharply greater wall width at the stem base (Figure 4). The latter feature compensates for the only modestly larger diameter at the base with respect to mid-height (see Figure 2 and Figure 3), due to earlier lignification [11], notwithstanding the maximum bending leverage occuring at the stem base [1,7]. A small decline in stem wall thickness was also observed over phenological stages in Tibet-A4, but this trend was only shown by the base segment in cultivar Ketos (Figure 4). Together, the reduction in diameter and wall thickness, particularly at the pulvinus, implies a decrease in the geometric component of bending resistance (*I*), even before substantial stem drying occurs. This small decrease in the geometrical component of bending strength can be expected to cause a correspondingly modest decline in *B_S_*.

**Figure 5:**
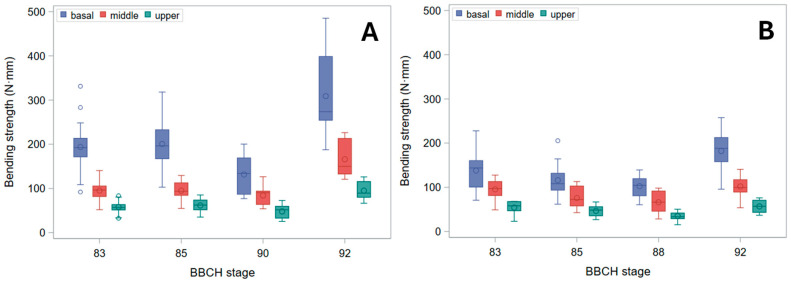
Bending strength (*B_S_*). Box plots for the basal, middle, and upper stem segments at subsequent phenological development stages (BBCH scale) are shown. (**A**) Cultivar Ketos. (**B**) Landrace Tibet-A4. Bending tests were carried out immediately after collecting at all stages but the last, for which *B_S_* was measured after further drying at RT for over five months.

**Figure 6:**
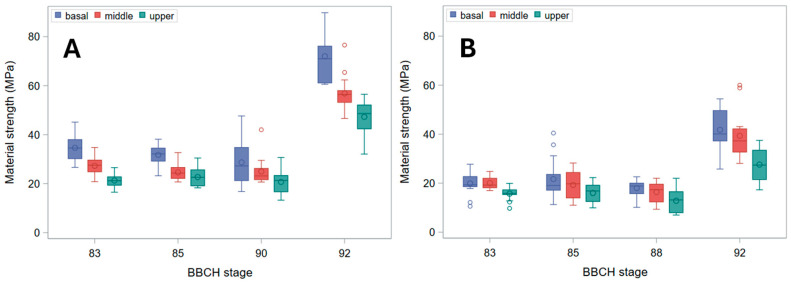
Material strength (σ_b_) just below the node. Box plots for the basal, middle, and upper stem segments at subsequent phenological development stages (BBCH scale) are shown. (**A**) Cultivar Ketos. (**B**) Landrace Tibet-A4.

**Figure 7:**
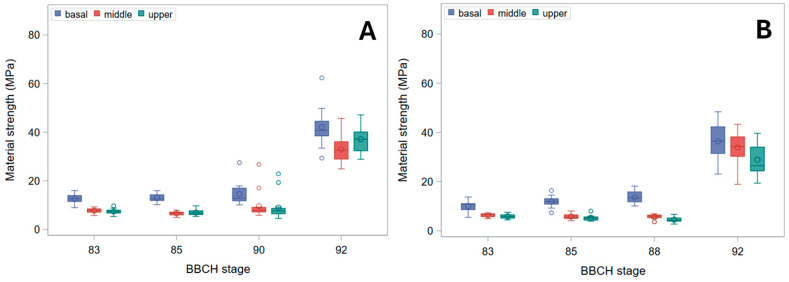
Material strength (σ_b_) at pulvinus. Box plots for the basal, middle, and upper stem segments at subsequent phenological development stages (BBCH scale) are shown. (**A**) Cultivar Ketos. (**B**) Landrace Tibet-A4.

Even *B_S_*, indeed, decreased from the basal to the upper stem segment (Figure 5). It also modestly declined through ripening, but ultimately rose sharply in harvest-ripe, dry culms. Biologically, this pattern identifies a distinct window during maturation—after physiological maturity but prior to full dry-down—when stems exhibit their lowest intrinsic resistance to bending. This transient mechanical weakness occurs despite plants still appearing structurally intact. Whereas the initial moderate decline through ripening was chiefly due to the reduction in diameter at pulvinus (Figure 3), the surge in fully senescent stems was caused by a strong increase in σ_b_ (Figure 6). Indeed, though σ_b_ (measured at the node) initially diminished during ripening, it markedly rose in dry stems (Figure 6). The decline in σ_b_, parallel to that in culm diameter and wall thickness during ripening, suggests that the observed reduction in bending strength is driven not only by geometry but also by a decrease in apparent σ_b_, consistent with a loss of turgor-related support in living tissues. When measured at the pulvinus, σ_b_ showed a similar pattern, but values were generally sharply lower (Figure 7), as expected [16], because pulvinus cell walls have limited secondary thickening, and pulvinus mechanical resistance therefore depends mainly on a larger diameter (compare Figure 2 and Figure 3) and higher turgor pressure [5].

As *B_S_* is the most interesting parameter for stem lodging in barley [16], the data summarized in Figure 5 were eventually subjected to analysis of variance (ANOVA). We presented raw data and did not apply ANOVA to other variables for three reasons. 1. Our primary aim was to elucidate the overarching trends in *B_S_* through ripening and maturation across genotypes and stem positions. 2. The sampling stages were not fully aligned between genotypes, rendering joint mean comparisons statistically invalid without additional assumptions that are explained in the next paragraph for *B_S_*. 3. Some variables require logarithmic (e.g., diameter [16]) or logit/probit transformation (moisture content percentage) for the analysis. In these cases, differences among means are better assessed on the transformed scale, because back-transformation produces asymmetric confidence intervals and can hide patterns that are clearer on the transformed scale. However, presenting many transformed values throughout the manuscript would obscure the biological meaning of the measurements and complicate interpretation, and evaluating the assumptions needed to resolve the problem described in point 2 would substantially increase analytical complexity. Hence, we performed targeted statistical analyses to justify the assumptions that allowed us to summarize the data in a manner fully suitable for capturing the overall trend in *B_S_* during ripening and maturation, which is the main aim of this study.

**Figure 8:**
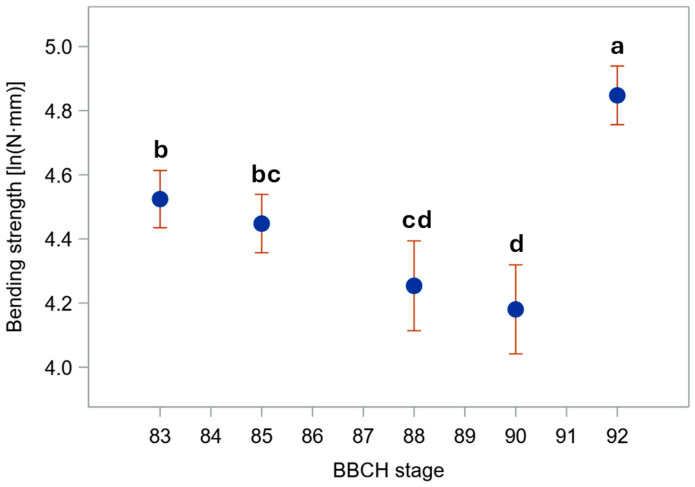
Bending strength (*B_S_*) through phenological development stages (BBCH scale) is shown on the natural logarithm scale, where statistical significance of effects was assessed under the assumption of negligible interactions. Values are averaged over genotypes and across the three positions at which stem segments were sampled. Whiskers represent 95% confidence intervals. Lower-case letters indicate significance of difference among BBCH stages: values with the same letter are not significantly different (*p* ≤ 0.05; SMM test). Bending tests were carried out immediately after collecting at all stages but the last, for which *B_S_* was measured after further drying at RT for over five months.

As expected [16], a first ANOVA (actually, a generalized linear mixed model) confirmed a consistent, highly significant difference in *B_S_* among stem segment positions (basal, middle, upper) at all stages for both genotypes (see Note 1 for Ketos and Tibet-A4 in Supplementary File ‘ANOVA Bs by genotype’). This analysis was conducted separately for each genotype (because, as noted above, the sampling stages were not fully aligned for the two genotypes) and included the fixed factors ‘segment position’, ‘stage’, and their interaction (see Note 1 in Supplementary File ‘ANOVA Bs by genotype’). A test of simple effects with multiple comparisons—that is, a test of the effects of the multiple levels of a factor (i.e., ‘segment position’) within each level of the other factor (i.e., ‘stage’)—was additionally included (see Notes 2 and 3 in Supplementary File ‘ANOVA Bs by genotype’) to specifically show that the *B_S_* values at basal, middle, and upper stem positions were all significantly different at each stage (in particular, their significances were always much higher than that of the interaction effect; compare the significances described in Note 2 with the significance of the interaction mentioned in Note 4 in Supplementary File ‘ANOVA Bs by genotype’). More importantly, the plot of their averages showed a consistent pattern across developmental stages (see Note 5 in Supplementary File ‘ANOVA Bs by genotype’). In other words, the ‘segment position’ × ‘stage’ interaction, even though significant (see Note 4 in Supplementary File ‘ANOVA Bs by genotype’), was small enough not to affect the rank order of *B_S_* for the different stem positions across stages (that is, the interaction effect was non-crossover). This means that changes in stem mechanical parameters can be followed over time by examining a single position, or they may be assessed by merging data over stem positions. This latter inference was used in the next step of the analysis: the data were re-analyzed (second ANOVA) by testing, for each genotype, changes in *B_S_* through stages, averaging over stem segment positions (Supplementary File ‘ANOVA Bs by genotype—averaged over positions’). To this aim this analysis included a post hoc multiple-comparisons test of the marginal means for factor ‘stage’—wherein, in practice, *B_S_* means at each developmental stage were obtained averaging across stem positions (see Note 1 in Supplementary File ‘ANOVA Bs by genotype—averaged over positions’). This approach retains the full factorial model (see Note 2 in Supplementary File ‘ANOVA Bs by genotype—averaged over positions’), but it uses the marginal means of BBCH stages to test overall differences among phenological stages while appropriately accounting for the segment position factor (see Notes 1 and 2 in Supplementary File ‘ANOVA Bs by genotype—averaged over positions’). This test confirmed the trend shown in Figure 5 for both genotypes (see Note 3 in Supplementary File ‘ANOVA Bs by genotype—averaged over positions’): *B_S_* diminishes during ripening and then surges in ripe, dry plants (actually, it showed that, in both genotypes, *B_S_* values at BBCH stages 88–90 were the smallest, significantly lower than at stages 83 and 92). As the trends of *B_S_* through developmental stages were very similar in both genotypes (see plots ‘LS-Means for Stage’ in Supplementary File ‘ANOVA Bs by genotype—averaged over positions’; commented on in Note 3), we merged data across genotypes to predict averages at all stages, under the assumption of additivity of the genotype effect (which implies no crossover interactions; that is, the effect is essentially the same across genotypes). We did this by performing a third ANOVA (Supplementary File ‘ANOVA Bs—averaged over genotypes and positions’) wherein the fixed factors ‘genotype’, ‘segment position’, and ‘stage’—but no interactions (most of which are non-estimable because different levels of ‘stage’ are entirely missing at different levels of ‘genotype’)—were included (see Note 1 in Supplementary File ‘ANOVA Bs—averaged over genotypes and positions’). Again, multiple comparisons of factor ‘stage’ marginal means were carried out (assuming all interactions are non-crossover), which in practice averages across stem positions and genotypes to obtain stage means (see Note 2 in Supplementary File ‘ANOVA Bs—averaged over genotypes and positions’). In this third ANOVA (the one Figure 8 is based on), effects of both ‘genotype’, stem ‘segment position’, and phenological ‘stage’ were shown to be significant (see Note 1 in Supplementary File ‘ANOVA Bs—averaged over genotypes and positions’), and the overall trend was supported (Figure 8), as already shown for each genotype separately (see plots ‘LS-Means for Stage’ in Supplementary File ‘ANOVA Bs by genotype—averaged over positions’; commented on in Note 3 in that file). As noted above, this approach requires that interaction effects—specifically, the ‘genotype’ × ‘stage’ effect and the three-way interaction—are non-crossover (that is, main factors’ effects are essentially additive), an assumption that was deemed reasonable as the trend is substantially consistent between the two genotypes (compare Note 3 in Supplementary File ‘ANOVA Bs—averaged over genotypes and positions’ with Note 3 for both genotypes in Supplementary File ‘ANOVA Bs by genotype—averaged over positions’), and the two differing sampling stages are quite close to each other, both temporally and physiologically. A study with more detailed sampling around physiological maturity (ideally, through BBCH stages 87–92) is, however, needed to confirm, in diverse genotypes, the exact stage at which *B_S_* reaches a minimum before rising again.

**Figure 9:**
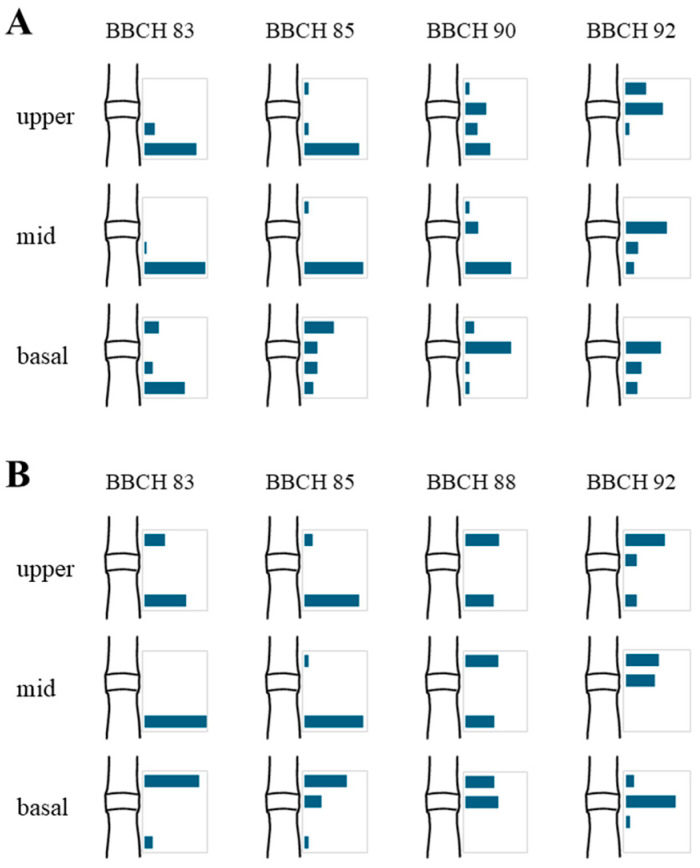
Bar plots of failure frequencies during the bending test for the basal, middle, and upper stem segments at subsequent phenological development stages (BBCH scale). In each plot, frequency of failure above the pulvinus, at the pulvinus, at the node, and below the node is shown. (**A**) Cultivar Ketos. (**B**) Landrace Tibet-A4.

Failure points and their frequencies were noted during the three-point bending test (Figure 9). Although the failure pattern at, or around, the barley node–pulvinus complex was not straightforward, two features emerge clearly from Figure 9: when a stem is bent, failure almost never occurs at the turgid pulvinus in living plants (BBCH stages 83–85), but it is much likely to occur at the withered pulvinus in dry plants and in plants that are drying out. These data confirm and support previous findings [16]. This shift in failure location marks a functional transition of the pulvinus from a mechanically reinforced structure in turgid plants to the primary point of weakness in senescent and drying stems, emphasizing its central role in late-stage lodging susceptibility.

As, in green plants, stem buckling does not occur at the pulvinus, which, instead, is the weak point for stem lodging resistance in ripe barley plants [16] (Figure 9), changes in *B_S_* should be imputed to the pulvinus during plant drying-down (BBCH stages 90 onward), but not before (when the pulvinus is turgid). To this regard, it is worth noting that the final increase observed for *B_S_* (Figure 5) was less pronounced than for σ_b_, whether the latter was measured at the node or the pulvinus (Figure 6 and Figure 7). For cultivar Ketos this was mostly due to the fact that, as σ_b_ was systematically lower at the pulvinus (compare Figure 6 with Figure 7), the shift to an overall lower level of σ_b_ at the later stage—when the pulvinus withers [16] and therefore becomes much more likely to fail (Figure 9)—curbs *B_S_* increase. In the case of landrace Tibet-A4, the surge in *B_S_* was, instead, largely restrained by a progressive reduction in the diameter of pulvinus, which was steeper than that observed for the node as well as with respect to cultivar Ketos (compare Figure 3 with Figure 2). The culm’s wall width showed a roughly similar trend whether measured at the node or the pulvinus, but when measured at the pulvinus the difference between the stem base and the two other positions was not as stark as when measured at the node (compare Figure 4 with Supplementary Figure S1). The area moment of inertia, *I*, displayed a pattern (Supplementary Figures S2 and S3) approximately similar to that of the diameter (see Figure 2 and Figure 3). When measured just below the node, *I* is the geometrical parameter that exhibits the most consistent decrease upward along the stem (Supplementary Figure S2). Although the culm base often has a larger cross-section to support the greater load, this is not always the case (Figure 2), because early lignification at the base can result in a diameter similar to that at mid-stem height. However, a wider culm-wall thickness at the base (Figure 4) usually more than compensates—in terms of *I*—for the reduced radial expansion caused by early lignification.

## 3. Discussion

Several structural gradients observed in this study—most notably the acropetal decline in culm diameter, wall thickness, and bending strength (Figure 2, Figure 3, Figure 4 and Figure 5), and, of course, the second moment of area (*I*, Supplementary Figure S2)—are consistent with established developmental patterns in barley stems [15,16,18]. The upward decrease in barley stem diameter reflects the typical developmental pattern of grass culms, which grow as a sequence of internodes that elongate telescopically from the base upward [3,15]. Each new internode forms inside the previous one and then extends outward like the sliding segments of a fishing pole. To this aim, each successive internode must be thinner than the one below it. Moreover, tissue properties undergo characteristic developmental changes, such as increased lignification, particularly at the stem base [11], so that material strength (σ_b_) shows a similar decreasing trend upward (Figure 6). These physiological effects match with the mechanical features required for the culm to support the ear once the culm is fully extended after heading: the soil-anchored stem is analogous to a vertical cantilever beam, with maximum bending moment at the fixed end (the stem base in grasses) [6], and bending strength (*B_S_*) decreases progressively from the bottom to the top of the stem (Figure 5), just as the bending moment does [5,16].

In addition to these features, the present study shows that further physiological effects affect *B_S_* during the maturation and drying stages: Figure 8 shows that *B_S_* declines during ripening, then surges to a maximum when the culms dry. The latter effect is due to the fact that material strength (σ_b_)—and thus *B_S_*—reaches a maximum when stem moisture falls below a threshold level [5]. Although dry barley stems are stiffer than moist and green ones [16], stem lodging tends to be more common for senescent plants close to harvest [1,7]. Indeed, ripe stems lose much of their mechanical strength if wetted, making lodging more likely when dry plants experience storms or persistent rainfalls [16]. Green plants, however, do not show a loss of mechanical strength of the culm even if they are moist, because their stiffness is supported by cell turgor [5].

**Figure 10:**
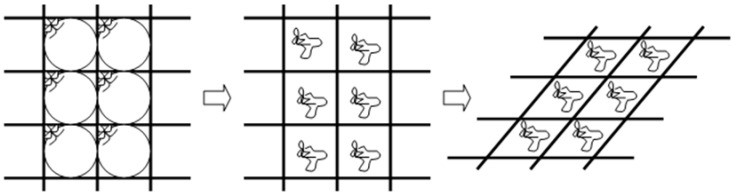
The stiffness of a plant tissue is due to both the material rigidity of cell walls (represented as lattice frame) and cell turgor (with the turgid plasmalemmas pushing against the cell walls represented as latex balloons). If the joints of the frame are hinged rather than fixed (that is, cell walls have some intrinsic rigidity, but they are not firm), the alveolar structure buckles when ballons deflate. Lignification is assumed to prevent this mode of buckling by making cell walls firmer.

On the one hand, therefore, green plants maintain a rigid stem in part because of turgor, since cells act as thin-walled balloons filled with pressurized fluid [8]. Figure 10 shows that tissues whose stiffness is mostly due to cell turgor buckle when turgor is lost. In living plant stems, therefore, *B_S_* is, in part, supported by axial tension from turgor pressure [5]. As remarked by Jarvis and McCann [8], indeed, rigidity of fully soft green tissues is caused by the internal pressure of living cells that pushes the plasmalemma against the cell wall—like it were the skin of a balloon (see Figure 10). In barley, which has a ‘hydro-skeleton’ [5] rather than fully soft green tissues, it has been estimated that turgor pressure accounts for about 10–40% of stem stiffness around heading, and the percentage gradually decreases thereafter [19], as internode tissues lignify [11].

Hozyo and Oda [20] observed that *B_S_* reaches a maximum two weeks after heading, and then it declines, possibly because of a decrease in turgor pressure. A moderate decline in *B_S_* as plants near maturity was observed in our experiment too (Figure 5). This was due to a reduction in diameter (Figure 2 and Figure 3) and, correspondingly, in *I* (Supplementary Figures S2 and S3), on which *B_S_* depends (Equation (2)). A small decrease in stem diameter, thickness of stem wall, and *I* at this stage was previously reported for rice [21], and it is expected to be associated with lower moisture content and turgor. Yet, in our study, this reduction was paralleled by a decrease in moisture content in Ketos but not in Tibet-A4 (Figure 1). Thus, even though culms shrank during ripening and senescence in both genotypes (Figure 2 and Figure 3), this trend did not match changes in water content across the first ripening stages (Figure 1), particularly for Tibet-A4. It is supposed that—at least in the greenhouse-grown well-watered plants used in this experiment—this initial mismatch was due to a decline in turgor caused by bulk flow export—the movement of water and dissolved solutes, such as soluble carbohydrates, amino acids, and ions, through the phloem from source to sink tissues—associated with the remobilization of reserves from the stem to the ear [22]. If water and dry matter were removed proportionally to their as-is composition, the water content would remain roughly the same while the stem gradually shrank. A reduction in turgor pressure can indeed occur even though water content does not decrease: during grain filling, carbohydrates, nitrogen compounds, and mineral nutrients present in the stem, sheaths, and leaves are remobilized to the developing grains, either through bulk flow export [22,23,24]—which can reduce turgor pressure without decreasing moisture content—or active phloem loading, which lowers osmotic pressure in the source tissues [25]. A decrease in stem dry matter was indeed observed in the study by Hozyo and Oda [26] in concomitance with the decline in *B_S_* and an increase in the dry matter of the ear. It is well known that, in barley stems, carbohydrate reserves (mainly fructans) reach a maximum at about BBCH stage 71 (grain watery ripe; approximately nine days after flowering), and then they decline substantially [3].

In our experiment, although plants showed typical acropetal yellowing (senescence) of leaves and culms during ripening, no substantial stem dry-down was observed until BBCH stage 90 (Figure 1), which is indeed the first stage of whole-plant senescence (complete loss of active metabolism and responsiveness) and post-ripening dry-down [17]. Preserving stem water during ripening can be useful for the plant to retain hydraulic conductance to support bulk flow export during reserves remobilization as long as possible—that is, until there is enough water in the soil/substrate—which was facilitated in our experiment by watering up to physiological maturity (BBCH stages 88–89). During ripening, therefore, *B_S_* slightly diminished up to BBCH stages 88–90 (Figure 5) owing to a decline in both *I* (Supplementary Figures S2 and S3) and the apparent σ_b_ (Figure 6 and Figure 7), presumably due to turgor loss (which is particularly evident as reduction in *I* at the pulvinus; Supplementary Figure S3).

On the other hand, when cereal plants dry down, equilibrating their moisture content with ambient air moisture, tissues become stiffer (Figure 5) and brittle [15,27] but more compliant if re-wetted [16]. In non-lignified plant tissues, in fact, hydration of cell walls that had previously dried greatly reduces their rigidity [28]. Dry walls are stiff due to tight hydrogen bonding and limited molecular mobility within cellulose–hemicellulose networks [28]. Upon rehydration, water penetrates the pectic matrix, disrupting these interactions and increasing molecular mobility and, thus, polymer flexibility and compliance, thereby lowering structural rigidity [28]. In general, extensive hydrogen bonding of water to wall polysaccharides softens the plant cell walls by reducing direct binding interactions between polysaccharides [10]. Hydration has little effect on the stiffness of cellulose microfibrils, which continue to behave as solid rods, but it profoundly alters the surrounding pectic matrix, which from a glass state expands into a rubbery, highly compliant gel hosting the stiff cellulose network [28,29,30,31]. Thus, water chiefly affects the mechanical properties of primary cell walls by acting on the pectic matrix, transforming dry tissues from rigid and brittle to soft and compliant [29,31]. This is the most likely reason for which dry barley stems—which are only partially lignified [11,12]—fail by buckling, whereas wet stems fail by creeping and become inflexible again when they re-dry [16].

It can be worth noting that the harvest-ripe samples (BBCH stage 92) were dried indoors to mimic the field final moisture state, because stem drying in the greenhouse differs from typical field drying owing to lower intensity of direct solar radiation, absence of wind exposure, and higher ambient humidity; therefore, the drying rate is much slower, and water content can remain significantly higher than in the field, which could introduce bias and reduce the observed *B_S_*. Although this procedure ensured moisture levels comparable to field harvest (see Figure 1), it might underestimate mechanical degradation associated with natural field drying; in particular, it prevented any re-humidification from rainfall or dew, which in field conditions can favor fungal activity and additional cell-wall degradation. In this respect, therefore, the values obtained here are more representative of physiological changes during maturation than of alterations caused by external agents, in accordance with the purpose of this study. The results for the final stage should thus be viewed as representing the mechanical properties of fully dried stems under controlled conditions, without weather-related or other exogenous interferences.

In this study, we, therefore, observed that, during barley maturation, stem resistance to bending shows noticeable dynamic changes: first, a slight, gradual decline—probably due to turgor decrease—and then, it rises to a maximum in fully ripe, dry plants (Figure 8). This pattern was investigated in stem segments including the pulvinus because it was previously shown that the pulvinus is a weak point in ripe barley plants with respect to the internode [16]. This is directly supported in this study by the increased frequency of failure at the pulvinus during bending tests in drying and dry plants (Figure 9), confirming that changes in *B_S_* during late maturation are disproportionately governed by pulvinus mechanics. Here, we measured the apparent material strength (σ_b_) through ripening and senescence stages, which integrates the contributions of both the true cell-wall material strength and turgor pressure [5]. Thus, as explained in the preceding discussion, the *B_S_* decline in the green stages mainly reflects turgor loss, whereas the increase in the drying stages reflects cell wall dehydration and stiffening. This shift is a consequence of the progressive transition of tissues from turgid to wilted and eventually to dry and inflexible, which traces back to the two completely different states of living and dry, dead cells.

## 4. Materials and Methods

### 4.1. Experiment Setup

Two barley genotypes, cultivar Ketos and landrace Tibet-A4 (described in [16]), were grown in a greenhouse at CREA—Research Centre for Genomics and Bioinformatics in Fiorenzuola d’Arda (Piacenza, Northern Italy) in 2024–2025. Both are six-row barleys; the former is winter-type and lodging-resistant, whereas the latter is spring-type and lodging-susceptible. Pots containing 3–4 plants were treated as independent experimental units and completely randomized, with a total of 16 pots per genotype. In a completely randomized design, genotypes are assigned at random to pots, giving each pot an equal probability of receiving any genotype. Seed was dressed with Redigo Pro (Bayer Crop Science, Monheim am Rhein, Germany). Each pot (square, 15 × 15 cm, 20 cm high) contained about 3.5 L of standard growing substrate (Special TNA 2; Vigorplant Italia srl, Fombio, Italy), which has medium-fine structure, pH 5.5–6.0, 90% total porosity, 280 kg/m^3^, electrical conductivity 0.25–0.35 dS/m (1:5 substrate:water extract), and included 1100 g/m^3^ of NPK 14-16-18 fertilizer plus micronutrients. Sowing was done in the last week of November. Ripening and senescence occurred from April through June, as is typical in Italy, with a slight anticipation in the greenhouse compared with outdoor field conditions, as is typical for unheated greenhouses. The average daily temperature in the greenhouse reached a minimum of about 2 °C in January and a maximum of 27 °C in June, and it never fell below 0 °C. This allowed winter-type cultivar Ketos to achieve vernalization, while spring-type landrace Tibet-A4 did not incur frost damage. A supplemental application of ammonium nitrate (~1 g per pot) was provided at tillering, and it corresponds to the nitrogen supply typically used in pot trials and sufficient to support growth up to maturity. Plants grew normally and did not show any nutrient-related limitations. Plastic stem supports were used to avoid bending of plant stems when handling the pots. Water was added 1–2 times a week during autumn and winter, and 3–5 times a week in springtime and up to physiological maturity (BBCH stages 88–89) by filling the pot saucer, whose low rim limited the water depth to about 2 cm.

Mechanical properties were measured at different phenological stages for each genotype, identified according to the BBCH development scale [17]. Stem segments about 13 cm long were collected from the stem starting from early dough stage (end of April) to harvest maturity (before mid-June). We aimed at collecting only the main culm of each plant, but when it was not immediately obvious which stem was the main one, the two strongest and tallest stems were collected. Three lengths were cut from each stem: a basal segment, centered at the lowest node for which a relatively straight-up segment could be obtained; a middle segment, centered at a node in the middle of the stem (from the base to the flag leaf node); and an upper segment, centered at the flag leaf node. Measurements and bending properties were immediately assessed after collecting each segment, at all stages but the last, for which the samples were further dried at room temperature (in paper envelopes) for over five months before testing because greenhouse conditions (no wind, attenuated sunlight, and higher air moisture due to many irrigated trials) prevented normal field drying.

Biometric measures (diameter and culm’s wall width, the latter determined as *r*_e_ − *r*_i_) were taken with a caliper (CoolantProof IP67 ABS digimatic caliper; Mitutoyo Italiana srl, Milano, Italy) at both the node and the pulvinus, and a three-point bending test was used to assess stem bending properties as described in [16]. Specifically, the node was centered on the bending support for testing and the loading force was manually applied with a fingertip and measured with a scale (on which the segment support was placed) [16]. Fifteen stems were assessed for each genotype and sampling time.

### 4.2. Calculations

In a three-point bending test with a mid-span load, the stem bending strength (*B_S_*) is calculated as the maximum bending moment at the point of maximum deflection [6]:

(3) *B_S_* = *F*_S_ × ℓ/4,

in which *F*_S_ is the measured maximum force that a stem portion can withstand while being bent, and ℓ is the span length of the stem segments laying between the supports (ℓ = 90 mm in our setup). As scales measure grams-force (gf), the readings must be transformed into kilograms-force (kgf) and multiplied times 9.80665—since 1 kgf ≡ 9.80665 Newtons (N)—to obtain *F*_S_ expressed in N.

Material strength (σ_b_) can, then, be calculated as the maximum material stress before failure [6] as

(4) σ_b_ = *B_S_* × *r*_e_/*I*

### 4.3. Statistical Analysis

Statistical analysis of *B_S_* data was generated using SAS^®^ software (software version 9.4, SAS/STAT version 15.3; SAS^®^ Studio release 3.82—SAS OnDemand for Academics; copyright © 2012–2023, SAS Institute Inc., Cary, NC, USA). Analysis of variance (ANOVA) was performed with the GLIMMIX procedure according to a generalized linear mixed model (GzLMM) assuming a lognormal distribution of the response variable [16]. About 15 culms were measured for each combination of genotype and BBCH stage. As sampling stage differed between genotypes—which makes overall means non-estimable at the differing stages without further assumptions—the first analysis was conducted separately for each genotype and included the fixed factors stem ‘segment position’, BBCH ‘stage’, and their interaction. The ‘stem’ effect was modeled as a random factor to account for the fact that three related measures were taken at different positions on the same subject (i.e., the same stem). Thus, this first analysis corresponds to a two-way mixed-effects ANOVA within a GzLMM framework, including the interaction

(5) log(*B_Sijk_*) = *μ* + Segment*_i_* + Stage*_j_* + (Segment × Stage)*_ij_* + Stem_*k*(*j*)_ + ε*_ijk_*,

where *μ* is the overall intercept, Segment*_i_* represents the fixed factor ‘segment position’ (with *i* = 1, 2, 3 levels), Stage*_j_* represents the fixed factor BBCH ‘stage’ (with *j* = 1, 2, 3, 4 levels), (Segment × Stage)*_ij_* is the interaction term, Stem_*k*(*j*)_ ~ N(0, σ^2^_stem_) represents the random factor ‘stem’ (with *k* ≈ 15 stems per each BBCH stage), and ε*_ijk_* ~ N(0, σ^2^) is the residual error. Stem_*k*(*j*)_ represents between-stem random variability at each stage (as different stems were necessarily sampled at each stage) and acts as a block within which the three levels of Segment*_i_* (namely, basal, middle, and upper segments) are associated. This specification assumes that the within-stem correlation among measurements taken at the different positions along the stem is adequately captured by a compound-symmetry covariance structure. All the factors were modeled as categorical variables.

The model was estimated with restricted maximum likelihood and optimized with dual quasi-Newton technique. The Kenward–Roger correction for the (prediction) standard errors and denominator degrees of freedom was applied. A test of simple effects was performed to test differences due to stem ‘segment position’ at each BBCH ‘stage’ by means of a partitioned analysis of the LS-means of the interaction ‘segment position’ × ‘stage’. For multiple comparisons of means, the Studentized Maximum Modulus (SMM) test for adjustment of *p*-values over all pairwise contrasts was applied. The output of this analysis is detailed in the Supplemetary file ‘ANOVA Bs by genotype’.

A second analysis was then performed—again separately for each genotype—on the same *B_S_* data and using the same GzLMM as in Equation (5). In this case, however, the inferential focus was shifted from the simple effects of ‘segment position’ to the main effect of ‘stage’. To do this, we estimated the LS-means of ‘stage’ averaged over segment position (i.e., the ‘stage’ marginal means) and applied an SMM correction for pairwise comparisons. In other words, a main-effect multiple-comparisons test of the marginal means of the ‘stage’ factor was included, so that data were de facto averaged over stem positions (keeping all the other model settings the same as in the first statistical model) to test overall differences among stages for each genotype. Using LS-means to average across levels of another factor while preserving the full model structure is standard in mixed-model inference and yields more accurate comparisons than those obtained by omitting factors from the model. The output of this analysis is detailed in the Supplementary File ‘ANOVA Bs by genotype—averaged over positions’.

A third statistical analysis of *B_S_* was then conducted to assess the main effects of the fixed factors ‘genotype’, ‘segment position’, and ‘stage’, and thereby to test for *B_S_* differences through phenological stages. The same lognormal GzLMM structure as before, including the random effect of ‘stem’, was retained, but all interaction terms among the fixed factors were excluded. This model corresponds to a three-way mixed-effects ANOVA within the GzLMM framework without interactions:

(6) log(*B_Sgijk_*) = *μ* + Genotype*_g_* + Segment*_i_* + Stage*_j_* + Stem_*g*(*jk*)_ + ε*_gijk_*,

where *μ* is the overall intercept, Genotype*_g_* (*g* = 1, 2) is the fixed ‘genotype’ effect, Segment*_i_* (*i* = 1, 2, 3) is the fixed ‘segment position’ effect, Stage*_j_* (*j* = 1, 2, 3, 4) is the fixed ‘stage’ effect. Stem_*g*(*jk*)_ ~ N(0, σ^2^_stem_) represents the random effect of ‘stem’ (with *k* ≈ 15 stems within each genotype × stage combination), and ε*_gijk_* ~ N(0, σ^2^) is the residual error. This analysis included multiple comparisons (adjusted with the SMM method) of the marginal means of the factor ‘stage’, which represent stage means averaged across genotypes and segment positions, under the assumption that interactions involving ‘stage’ are absent (i.e., no crossover interactions). The output of this analysis is detailed in the Supplementary File ‘ANOVA Bs—averaged over genotypes and positions’.

## 5. Conclusions

Our study demonstrates that—in the absence of rain, which would otherwise compromise the culm’s mechanical strength in harvest-ripe plants—the bending strength (*B_S_*) associated with the physiological condition of the plant is highest when the crop is fully ripe but it has not yet dried down to a water content suitable for harvesting (that is, around BBCH stage 89). Thus, by explicitly linking temporal changes in stem geometry, material strength, and failure location, our results delineate a narrow but critical physiological window during which barley stems are mechanically weakest. However, it is known that the actual lodging risk increases toward harvest, as this period typically coincides with seasonal storms in most barley-growing climates, and dry straw softens and bends more readily when wet.

The fact that culm bending resistance reaches its minimum between physiological maturity and full harvest dryness suggests that barley breeding should target traits that maintain geometric and material strength specifically late in maturation, when turgor is declining and culm moisture remains high. As our study shows that both the area moment of inertia (*I*) and material strength (σ_b_) decline during ripening (but before drying), breeders could advantageously select for genotypes with stronger turgor-independent mechanical strength, for example, by promoting enhanced secondary-wall deposition before ripening, that is, further reinforcing the barley ‘hydro-skeleton’. Since fully dry stems regain high rigidity but become weaker if re-wetted, selection could also prioritize genotypes whose mechanical strength is less moisture-sensitive, reducing the drop in stiffness after rain. Altogether, meaningful targets for breeders are genotypes that: (i) have larger diameters and thicker walls that decline less across maturation; (ii) possess a cell wall composition that retains adequate stiffness even after the straw has dried and been re-wetted; and, specifically, (iii) maintain high σ_b_ during the ripening-to-drying transition.

## Data Availability

Data is contained in Supplementary File Dataset.xlsx.

## Supplementary Materials

The following supporting information can be downloaded at https://www.mdpi.com/article/10.3390/plants15081234/s1. Supplementary Figures S1–S3—Figure S1: Culm wall thickness (*r*_e_ − *r*_i_) at pulvinus; Figure S2: Area moment of inertia (*I*) just below the node; Figure S3: Area moment of inertia (*I*) at pulvinus; html files ‘ANOVA Bs by genotype’, ‘ANOVA Bs by genotype—averaged over positions’, and ‘ANOVA Bs—averaged over genotypes and positions’ detail results of statistical analyses; xlsx file ‘Dataset’ provide data used for statistical analysis.

## Abbreviations

The following abbreviations are used in this manuscript:

ANOVA	Analysis of Variance
RT	Room Temperature
