# Supplementary material for: Barley Stem Bending Resistance Declines During Maturation, Then Peaks in Ripe, Dry Plants
Source: Plants (Basel). 2026 Apr 17;15(8):1234. doi: 10.3390/plants15081234 (PMC13119592; doi:10.3390/plants15081234)
Supplement: Supplementary file 1 [file plants-15-01234-s001.zip › ANOVA Bs - averaged over genotypes and positions.pdf]

The GLIMMIX Procedure

This block summarizes the GLIMMIX model specification (distribution, link, estimation method). It is provided for completeness and reproducibility.

Model Information	
**Data Set**	WORK.IMPORT
**Response Variable**	Bs
**Response Distribution**	Lognormal
**Link Function**	Identity
**Variance Function**	Default
**Variance Matrix**	Not blocked
**Estimation Technique**	Restricted Maximum Likelihood
**Degrees of Freedom Method**	Kenward-Roger2
**Fixed Effects SE Adjustment**	Kenward-Roger2

This block summarizes the fixed and random effects structure.

Class Level Information		
**Class**	**Levels**	**Values**
**Genotype**	2	Ketos Tibet
**Position**	3	basal middle upper
**Stage**	5	83 85 88 90 92
**stem**	15	1 2 3 4 5 6 7 8 9 10 11 12 13 14 15

**Number of Observations Read**	405
**Number of Observations Used**	395

Dimensions	
**G-side Cov. Parameters**	1
**R-side Cov. Parameters**	1
**Columns in X**	11
**Columns in Z**	120
**Subjects (Blocks in V)**	1
**Max Obs per Subject**	395

Optimization Information	
**Optimization Technique**	Dual Quasi-Newton
**Parameters in Optimization**	1
**Lower Boundaries**	1
**Upper Boundaries**	0
**Fixed Effects**	Profiled
**Residual Variance**	Profiled
**Starting From**	Data

The GLIMMIX Procedure

Iteration History					
**Iteration**	**Restarts**	**Evaluations**	**Objective Function**	**Change**	**Max Gradient**
**0**	**0**	4	31.984429673	.	30.3068
**1**	**0**	2	24.467141717	7.51728796	4.232727
**2**	**0**	4	24.140230401	0.32691132	0.005427
**3**	**0**	2	24.140229755	0.00000065	0.000767
**4**	**0**	2	24.140229742	0.00000001	1.678E-7

Convergence criterion (GCONV=1E-8) satisfied.

The Fit Statistics table summarizes how well the specified model describes the data. It includes likelihood-based criteria used to compare alternative models: lower values indicate a better fit, penalizing excessive model complexity. The table also include the Generalized Chi-Square/DF statistics, which indicates whether the model adequately accounts for overdispersion. Overall, these statistics help assess the adequacy of the model and guide model comparison.

Fit Statistics	
**-2 Res Log Likelihood**	24.14
**AIC (smaller is better)**	28.14
**AICC (smaller is better)**	28.17
**BIC (smaller is better)**	33.72
**CAIC (smaller is better)**	35.72
**HQIC (smaller is better)**	30.40
**Generalized Chi-Square**	13.08
**Gener. Chi-Square / DF**	0.03

This table provides information on the model's random effects structure. The covariance parameter estimates quantify variability among stems and residual variance in the mixed model.

Covariance Parameter Estimates		
**Cov Parm**	**Estimate**	**Standard Error**
**stem(Genotype*Stage)**	0.05203	0.008515
**Residual**	0.03381	0.002910

This table reports the significance of the fixed factors (Genotype, Position, Stage) in the GLIMMIX model.
A significant main effect indicates that least squares means differ among the levels of that factor.

Type III Tests of Fixed Effects				
**Effect**	**Num DF**	**Den DF**	**F Value**	**Pr > F**
**Genotype**	1	109.8	41.62	<.0001
**Stage**	4	111.5	24.05	<.0001
**Position**	2	271.2	1136.86	<.0001

Note 1:
This is the ANOVA table. Three fixed factors, but no interactions, are included in this model. Significances of effects are highlighted.

The GLIMMIX Procedure

This table reports estimates of LS-means (on the log scale) for each Stage. Significance levels correspond to differences from zero; that is, means are tested for the null value hypothesis.

Stage Least Squares Means								
Stage	Estimate	Standard Error	DF	t Value	Pr > |t|	Alpha	Lower	Upper
83	4.5241	0.04495	102.4	100.66	<.0001	0.05	4.4349	4.6132
85	4.4478	0.04593	112.3	96.83	<.0001	0.05	4.3568	4.5388
88	4.2538	0.07080	116.1	60.09	<.0001	0.05	4.1136	4.3940
90	4.1804	0.07011	112	59.62	<.0001	0.05	4.0414	4.3193
92	4.8477	0.04620	114.7	104.94	<.0001	0.05	4.7562	4.9392

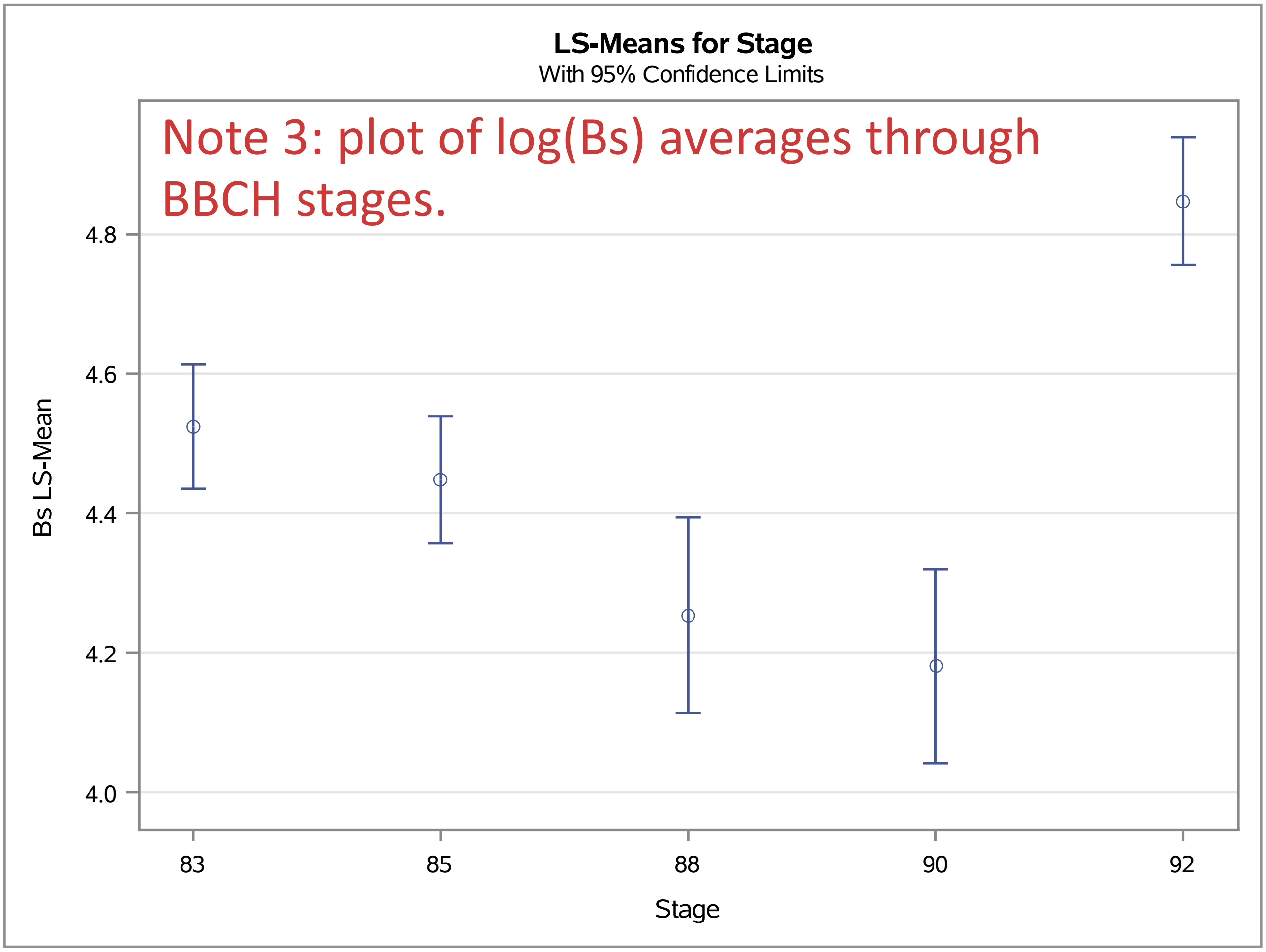

Note 2: multiple comparisons of Bs marginal means among different BBCH stages (below).

This table reports pairwise comparisons between Stages, with multiplicity correction (SMM). Positive estimates indicate that the first Stage listed has a higher mean than the second.

Differences of Stage Least Squares Means Adjustment for Multiple Comparisons: SMM												
Stage	Stage	Estimate	Standard Error	DF	t Value	Pr > |t|	Adj P	Alpha	Lower	Upper	Adj Lower	Adj Upper
83	85	0.07630	0.06427	107.3	1.19	0.2377	0.9285	0.05	-0.05109	0.2037	-0.1072	0.2598
83	88	0.2703	0.08425	114.2	3.21	0.0017	0.0171	0.05	0.1034	0.4372	0.03002	0.5106
83	90	0.3437	0.08287	106.9	4.15	<.0001	0.0007	0.05	0.1794	0.5080	0.1071	0.5804
83	92	-0.3236	0.06444	108.4	-5.02	<.0001	<.0001	0.05	-0.4513	-0.1959	-0.5076	-0.1396
85	88	0.1940	0.08439	115	2.30	0.0233	0.2071	0.05	0.02684	0.3612	-0.04664	0.4346
85	90	0.2674	0.08382	112.1	3.19	0.0018	0.0182	0.05	0.1013	0.4335	0.02829	0.5065
85	92	-0.3999	0.06515	113.5	-6.14	<.0001	<.0001	0.05	-0.5290	-0.2709	-0.5857	-0.2141

The GLIMMIX Procedure

Differences of Stage Least Squares Means
Adjustment for Multiple Comparisons: SMM

Stage	Stage	Estimate	Standard Error	DF	t Value	Pr > |t|	Adj P	Alpha	Lower	Upper	Adj Lower	Adj Upper
88	90	0.07342	0.1064	113.5	0.69	0.4915	0.9987	0.05	-0.1373	0.2842	-0.2300	0.3769
88	92	-0.5939	0.08461	116.1	-7.02	<.0001	<.0001	0.05	-0.7615	-0.4263	-0.8351	-0.3527
90	92	-0.6673	0.08387	112.3	-7.96	<.0001	<.0001	0.05	-0.8335	-0.5012	-0.9066	-0.4281
