# Supplementary material for: Barley Stem Bending Resistance Declines During Maturation, Then Peaks in Ripe, Dry Plants
Source: Plants (Basel). 2026 Apr 17;15(8):1234. doi: 10.3390/plants15081234 (PMC13119592; doi:10.3390/plants15081234)
Supplement: Supplementary file 1 [file plants-15-01234-s001.zip › ANOVA Bs by genotype - averaged over positions.pdf]

The GLIMMIX Procedure

Genotype=Ketos

This block summarizes the GLIMMIX model specification (distribution, link, estimation method). It is provided for completeness and reproducibility.

Model Information	
Data Set	WORK.IMPORT
Response Variable	Bs
Response Distribution	Lognormal
Link Function	Identity
Variance Function	Default
Variance Matrix	Not blocked
Estimation Technique	Restricted Maximum Likelihood
Degrees of Freedom Method	Kenward-Roger2
Fixed Effects SE Adjustment	Kenward-Roger2

This block summarizes the fixed and random effects structure.

Class Level Information		
Class	Levels	Values
Genotype	1	Ketos
Position	3	basal middle upper
Stage	4	83 85 90 92
stem	15	1 2 3 4 5 6 7 8 9 10 11 12 13 14 15

Number of Observations Read	225
Number of Observations Used	224

Dimensions	
G-side Cov. Parameters	1
R-side Cov. Parameters	1
Columns in X	20
Columns in Z	60
Subjects (Blocks in V)	1
Max Obs per Subject	224

Optimization Information	
Optimization Technique	Dual Quasi-Newton
Parameters in Optimization	1
Lower Boundaries	1
Upper Boundaries	0
Fixed Effects	Profiled
Residual Variance	Profiled
Starting From	Data

The GLIMMIX Procedure

Genotype=Ketos

Iteration History

Iteration	Restarts	Evaluations	Objective Function	Change	Max Gradient
0	0	4	-9.86071295	.	21.80052
1	0	2	-15.12096023	5.26024728	2.944894
2	0	4	-15.36332641	0.24236617	0.162319
3	0	2	-15.36423308	0.00090667	0.027244
4	0	2	-15.36425971	0.00002663	0.000316
5	0	2	-15.36425972	0.00000000	6.234E-7

Convergence criterion (GCONV=1E-8) satisfied.

The Fit Statistics table summarizes how well the specified model describes the data. It includes likelihood-based criteria used to compare alternative models: lower values indicate a better fit, penalizing excessive model complexity. The table also include the Generalized Chi-Square/DF statistics, which indicates whether the model adequately accounts for overdispersion. Overall, these statistics help assess the adequacy of the model and guide model comparison.

Fit Statistics

-2 Res Log Likelihood	-15.36
AIC (smaller is better)	-11.36
AICC (smaller is better)	-11.31
BIC (smaller is better)	-7.18
CAIC (smaller is better)	-5.18
HQIC (smaller is better)	-9.73
Generalized Chi-Square	6.11
Gener. Chi-Square / DF	0.03

This table provides information on the model's random effects structure. The covariance parameter estimates quantify variability among stems and residual variance in the mixed model.

Covariance Parameter Estimates

Cov Parm	Estimate	Standard Error
stem(Stage)	0.04018	0.009395
Residual	0.02884	0.003286

This table reports the significance of the fixed factors (Position, Stage) and their interaction in the GLIMMIX model.
A significant main effect indicates that least squares means differ among the levels of that factor. A significant interaction indicates that the effect of Position varies across Stages (or vice versa).

Type III Tests of Fixed Effects

Effect	Num DF	Den DF	F Value	Pr > F
Position	2	154.1	773.03	<.0001
Stage	3	54.13	30.17	<.0001
Position*Stage	6	154.1	2.99	0.0086

Note 2 (Ketos):
This is the ANOVA table. The two factors and their interaction are the same as in the first analysis.

The GLIMMIX Procedure

Genotype=Ketos

This table reports estimates of LS-means (on the log scale) for each Position within each Stage. Significance levels correspond to differences from zero; that is, means are tested for the null value hypothesis.

Stage Least Squares Means

Stage	Estimate	Standard Error	DF	t Value	Pr > |t|	Alpha	Lower	Upper
83	4.6018	0.05480	46.48	83.97	<.0001	0.05	4.4915	4.7121
85	4.6292	0.05761	57	80.35	<.0001	0.05	4.5138	4.7445
90	4.3506	0.05761	57	75.51	<.0001	0.05	4.2352	4.4659
92	5.1077	0.05761	57	88.66	<.0001	0.05	4.9924	5.2231

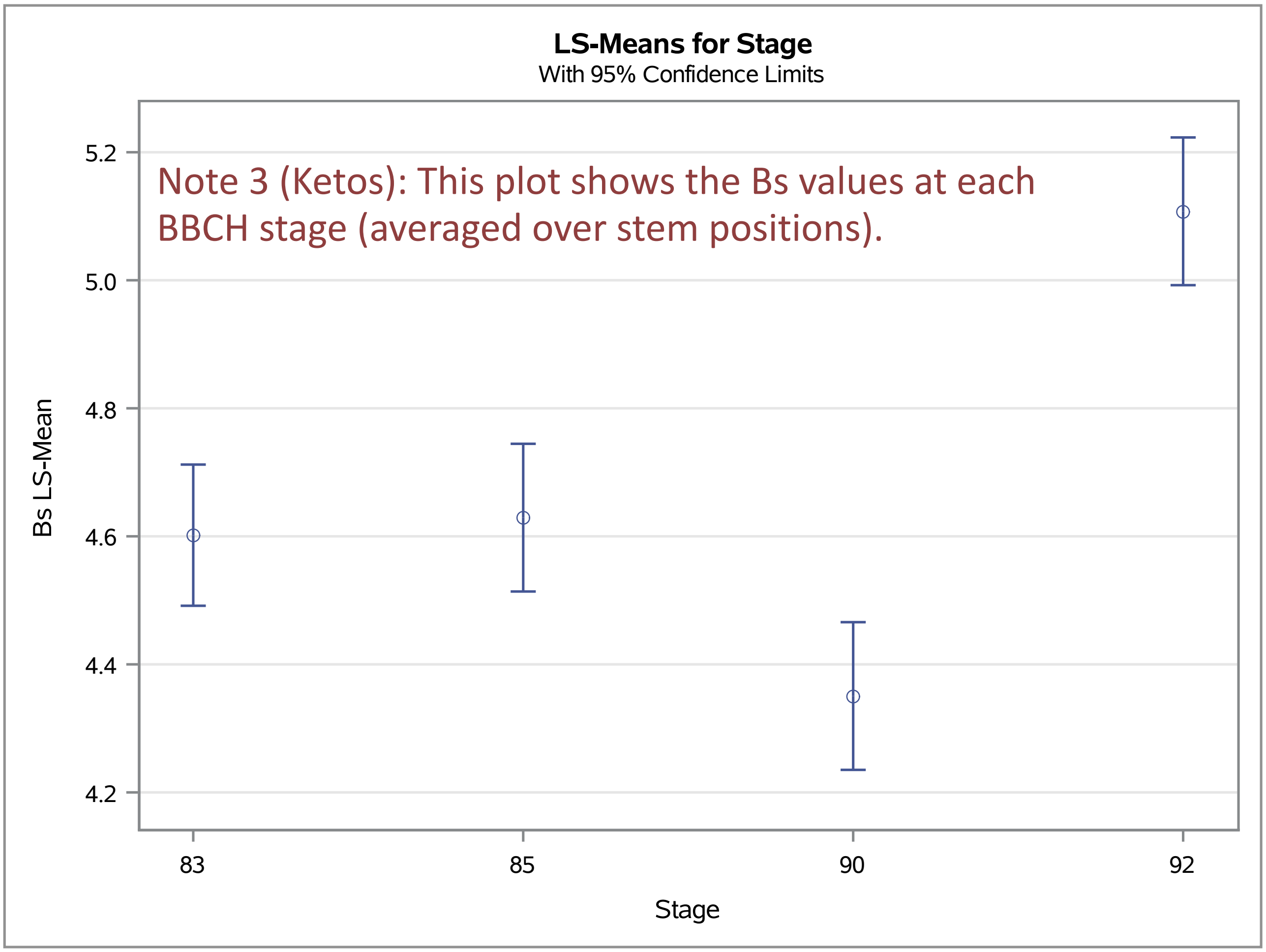

This table reports pairwise comparisons between Stages, with multiplicity correction (SMM). Positive estimates indicate that the first Stage listed has a higher mean than the second.

Differences of Stage Least Squares Means
Adjustment for Multiple Comparisons: SMM

Stage	Stage	Estimate	Standard Error	DF	t Value	Pr > |t|	Adj P	Alpha	Lower	Upper	Adj Lower	Adj Upper
83	85	-0.02736	0.07951	51.65	-0.34	0.7321	0.9996	0.05	-0.1869	0.1322	-0.2445	0.1897
83	90	0.2512	0.07951	51.65	3.16	0.0026	0.0156	0.05	0.09165	0.4108	0.03415	0.4683
83	92	-0.5059	0.07951	51.65	-6.36	<.0001	<.0001	0.05	-0.6655	-0.3464	-0.7230	-0.2889
85	90	0.2786	0.08148	57	3.42	0.0012	0.0069	0.05	0.1154	0.4418	0.05693	0.5003
85	92	-0.4786	0.08148	57	-5.87	<.0001	<.0001	0.05	-0.6417	-0.3154	-0.7003	-0.2569
90	92	-0.7572	0.08148	57	-9.29	<.0001	<.0001	0.05	-0.9203	-0.5940	-0.9789	-0.5355

Note 1 (Ketos): The table above shows the pair-wise multiple-comparisons test of the marginal means at different BBCH stages. Significances of differences are highlighted.

The GLIMMIX Procedure

Genotype=Tibet

Model Information	
**Data Set**	WORK.IMPORT
**Response Variable**	Bs
**Response Distribution**	Lognormal
**Link Function**	Identity
**Variance Function**	Default
**Variance Matrix**	Not blocked
**Estimation Technique**	Restricted Maximum Likelihood
**Degrees of Freedom Method**	Kenward-Roger2
**Fixed Effects SE Adjustment**	Kenward-Roger2

Class Level Information		
**Class**	**Levels**	**Values**
**Genotype**	1	Tibet
**Position**	3	basal middle upper
**Stage**	4	83 85 88 92
**stem**	15	1 2 3 4 5 6 7 8 9 10 11 12 13 14 15

**Number of Observations Read**	180
**Number of Observations Used**	171

Dimensions	
**G-side Cov. Parameters**	1
**R-side Cov. Parameters**	1
**Columns in X**	20
**Columns in Z**	60
**Subjects (Blocks in V)**	1
**Max Obs per Subject**	171

Optimization Information	
**Optimization Technique**	Dual Quasi-Newton
**Parameters in Optimization**	1
**Lower Boundaries**	1
**Upper Boundaries**	0
**Fixed Effects**	Profiled
**Residual Variance**	Profiled
**Starting From**	Data

The GLIMMIX Procedure

Genotype=Tibet

Iteration History

Iteration	Restarts	Evaluations	Objective Function	Change	Max Gradient
0	0	4	33.517357318	.	2.538447
1	0	2	33.229944526	0.28741279	0.982819
2	0	2	33.143637358	0.08630717	0.230616
3	0	2	33.139288692	0.00434867	0.029872
4	0	2	33.139217402	0.00007129	0.000778
5	0	2	33.139217353	0.00000005	2.544E-6

Convergence criterion (GCONV=1E-8) satisfied.

Fit Statistics

-2 Res Log Likelihood	33.14
AIC (smaller is better)	37.14
AICC (smaller is better)	37.22
BIC (smaller is better)	41.33
CAIC (smaller is better)	43.33
HQIC (smaller is better)	38.78
Generalized Chi-Square	4.80
Gener. Chi-Square / DF	0.03

Covariance Parameter Estimates

Cov Parm	Estimate	Standard Error
stem(Stage)	0.06133	0.01382
Residual	0.03018	0.004220

Type III Tests of Fixed Effects

Effect	Num DF	Den DF	F Value	Pr > F
Position	2	103.4	464.14	<.0001
Stage	3	55.47	9.72	<.0001
Position*Stage	6	103.3	2.69	0.0182

Note 2 (Tibet):
This is the ANOVA table. The two factors and their interaction are the same as in the first analysis.

The GLIMMIX Procedure

Genotype=Tibet

Stage Least Squares Means								
Stage	Estimate	Standard Error	DF	t Value	Pr > |t|	Alpha	Lower	Upper
83	4.4574	0.06915	54.61	64.46	<.0001	0.05	4.3188	4.5960
85	4.2664	0.06899	54.13	61.84	<.0001	0.05	4.1281	4.4047
88	4.0805	0.06981	56.59	58.45	<.0001	0.05	3.9407	4.2203
92	4.5752	0.06981	56.59	65.54	<.0001	0.05	4.4354	4.7150

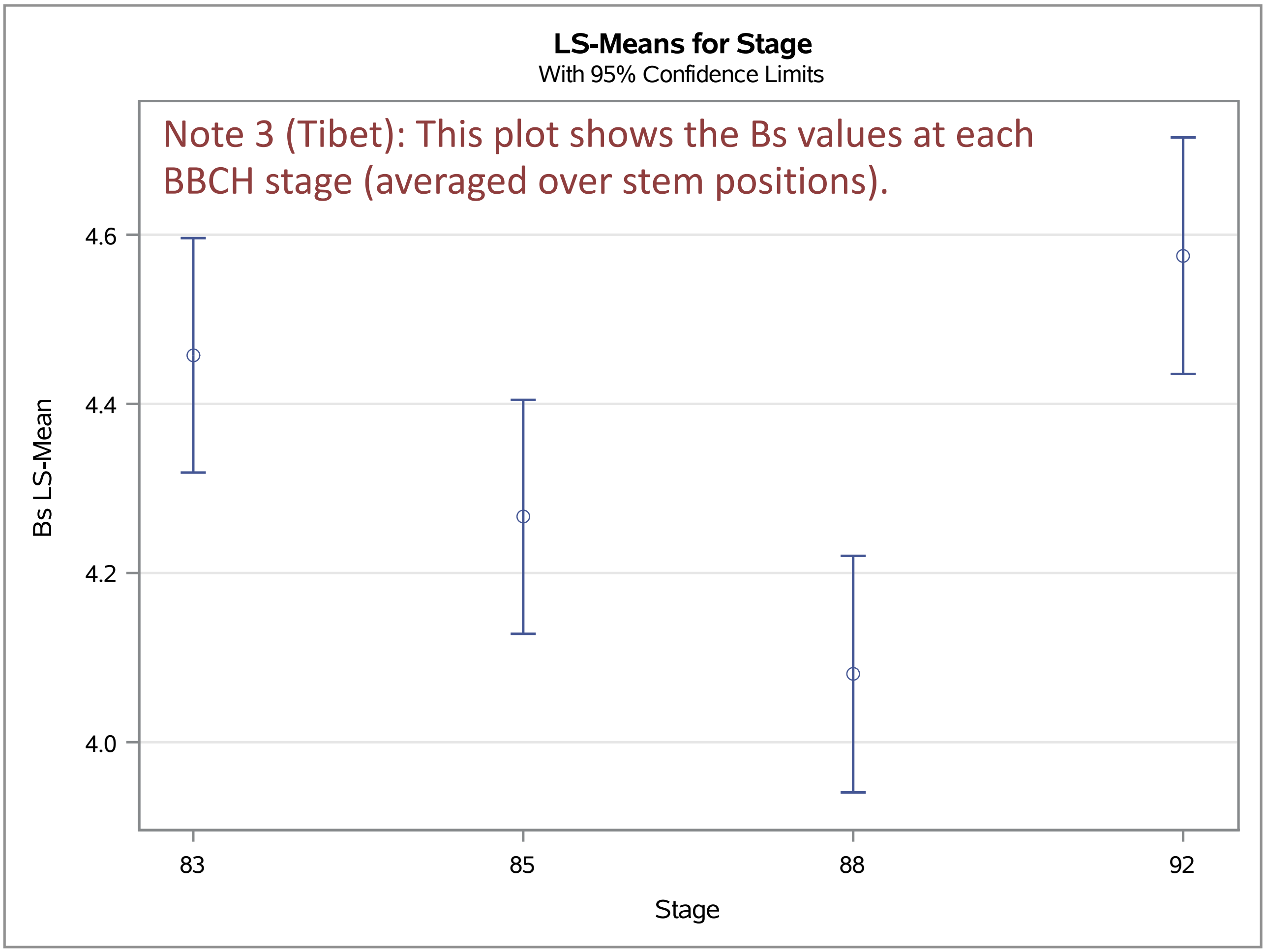

Differences of Stage Least Squares Means<br>Adjustment for Multiple Comparisons: SMM												
Stage	Stage	Estimate	Standard Error	DF	t Value	Pr > |t|	Adj P	Alpha	Lower	Upper	Adj Lower	Adj Upper
83	85	0.1910	0.09768	54.37	1.96	0.0557	0.2844	0.05	-0.00481	0.3868	-0.07519	0.4572
83	88	0.3769	0.09826	55.6	3.84	0.0003	0.0019	0.05	0.1800	0.5738	0.1094	0.6445
83	92	-0.1178	0.09826	55.6	-1.20	0.2356	0.7906	0.05	-0.3147	0.07906	-0.3854	0.1498
85	88	0.1859	0.09815	55.36	1.89	0.0634	0.3177	0.05	-0.01073	0.3826	-0.08136	0.4532
85	92	-0.3088	0.09815	55.36	-3.15	0.0027	0.0157	0.05	-0.5055	-0.1121	-0.5761	-0.04151
88	92	-0.4947	0.09873	56.59	-5.01	<.0001	<.0001	0.05	-0.6925	-0.2970	-0.7634	-0.2261

Note 1 (Tibet): The table above shows the pair-wise multiple-comparisons test of the marginal means at different BBCH stages. Significances of differences are highlighted.
