# Supplementary material for: Barley Stem Bending Resistance Declines During Maturation, Then Peaks in Ripe, Dry Plants
Source: Plants (Basel). 2026 Apr 17;15(8):1234. doi: 10.3390/plants15081234 (PMC13119592; doi:10.3390/plants15081234)
Supplement: Supplementary file 1 [file plants-15-01234-s001.zip › ANOVA Bs by genotype.pdf]

The GLIMMIX Procedure

Genotype=Ketos

This block summarizes the GLIMMIX model specification (distribution, link, estimation method). It is provided for completeness and reproducibility.

Model Information	
**Data Set**	WORK.IMPORT
**Response Variable**	Bs
**Response Distribution**	Lognormal
**Link Function**	Identity
**Variance Function**	Default
**Variance Matrix**	Not blocked
**Estimation Technique**	Restricted Maximum Likelihood
**Degrees of Freedom Method**	Kenward-Roger2
**Fixed Effects SE Adjustment**	Kenward-Roger2

This block summarizes the fixed and random effects structure.

Class Level Information		
**Class**	**Levels**	**Values**
**Genotype**	1	Ketos
**Position**	3	basal middle upper
**Stage**	4	83 85 90 92
**stem**	15	1 2 3 4 5 6 7 8 9 10 11 12 13 14 15

**Number of Observations Read**	225
**Number of Observations Used**	224

Dimensions	
**G-side Cov. Parameters**	1
**R-side Cov. Parameters**	1
**Columns in X**	20
**Columns in Z**	60
**Subjects (Blocks in V)**	1
**Max Obs per Subject**	224

Optimization Information	
**Optimization Technique**	Dual Quasi-Newton
**Parameters in Optimization**	1
**Lower Boundaries**	1
**Upper Boundaries**	0
**Fixed Effects**	Profiled
**Residual Variance**	Profiled
**Starting From**	Data

The GLIMMIX Procedure

Genotype=Ketos

Iteration History					
**Iteration**	**Restarts**	**Evaluations**	**Objective Function**	**Change**	**Max Gradient**
**0**	**0**	4	-9.86071295	.	21.80052
**1**	**0**	2	-15.12096023	5.26024728	2.944894
**2**	**0**	4	-15.36332641	0.24236617	0.162319
**3**	**0**	2	-15.36423308	0.00090667	0.027244
**4**	**0**	2	-15.36425971	0.00002663	0.000316
**5**	**0**	2	-15.36425972	0.00000000	6.234E-7

Convergence criterion (GCONV=1E-8) satisfied.

The Fit Statistics table summarizes how well the specified model describes the data. It includes likelihood-based criteria used to compare alternative models: lower values indicate a better fit, penalizing excessive model complexity. The table also include the Generalized Chi-Square/DF statistics, which indicates whether the model adequately accounts for overdispersion. Overall, these statistics help assess the adequacy of the model and guide model comparison.

Fit Statistics	
**-2 Res Log Likelihood**	-15.36
**AIC (smaller is better)**	-11.36
**AICC (smaller is better)**	-11.31
**BIC (smaller is better)**	-7.18
**CAIC (smaller is better)**	-5.18
**HQIC (smaller is better)**	-9.73
**Generalized Chi-Square**	6.11
**Gener. Chi-Square / DF**	0.03

This table provides information on the model’s random effects structure. The covariance parameter estimates quantify variability among stems and residual variance in the mixed model.

Covariance Parameter Estimates		
**Cov Parm**	**Estimate**	**Standard Error**
**stem(Stage)**	0.04018	0.009395
**Residual**	0.02884	0.003286

This table reports the significance of the fixed factors (Position, Stage) and their interaction in the GLIMMIX model. A significant main effect indicates that least squares means differ among the levels of that factor. A significant interaction indicates that the effect of Position varies across Stages (or vice versa).

Type III Tests of Fixed Effects				
**Effect**	**Num DF**	**Den DF**	**F Value**	**Pr > F**
**Position**	2	154.1	773.03	<.0001
**Stage**	3	54.13	30.17	<.0001
**Position*Stage**	6	154.1	2.99	0.0086

Note 1 (Ketos):
This is the ANOVA table.
The stem segment 'Positions' factor has a highly significant effect.

Note 4 (Ketos):
This is the ANOVA table.
The interaction effect 'Position' x 'Stage' is signficant, but not < 0.0001.

The GLIMMIX Procedure

Genotype=Ketos

This table reports estimates of LS-means (on the log scale) for each Position within each Stage. Significance levels correspond to differences from zero; that is, means are tested for the null value hypothesis.

Position*Stage Least Squares Means

Position	Stage	Estimate	Standard Error	DF	t Value	Pr > |t|	Alpha	Lower	Upper
basal	83	5.2392	0.06033	68.35	86.84	<.0001	0.05	5.1188	5.3595
basal	85	5.2680	0.06783	104	77.67	<.0001	0.05	5.1335	5.4025
basal	90	4.8302	0.06783	104	71.21	<.0001	0.05	4.6957	4.9648
basal	92	5.6973	0.06783	104	83.99	<.0001	0.05	5.5628	5.8318
middle	83	4.5352	0.06033	68.35	75.17	<.0001	0.05	4.4148	4.6555
middle	85	4.5307	0.06783	104	66.80	<.0001	0.05	4.3962	4.6652
middle	90	4.4059	0.06783	104	64.96	<.0001	0.05	4.2714	4.5404
middle	92	5.0886	0.06783	104	75.02	<.0001	0.05	4.9541	5.2231
upper	83	4.0311	0.06065	69.72	66.46	<.0001	0.05	3.9101	4.1520
upper	85	4.0887	0.06783	104	60.28	<.0001	0.05	3.9542	4.2232
upper	90	3.8155	0.06783	104	56.25	<.0001	0.05	3.6810	3.9500
upper	92	4.5374	0.06783	104	66.89	<.0001	0.05	4.4028	4.6719

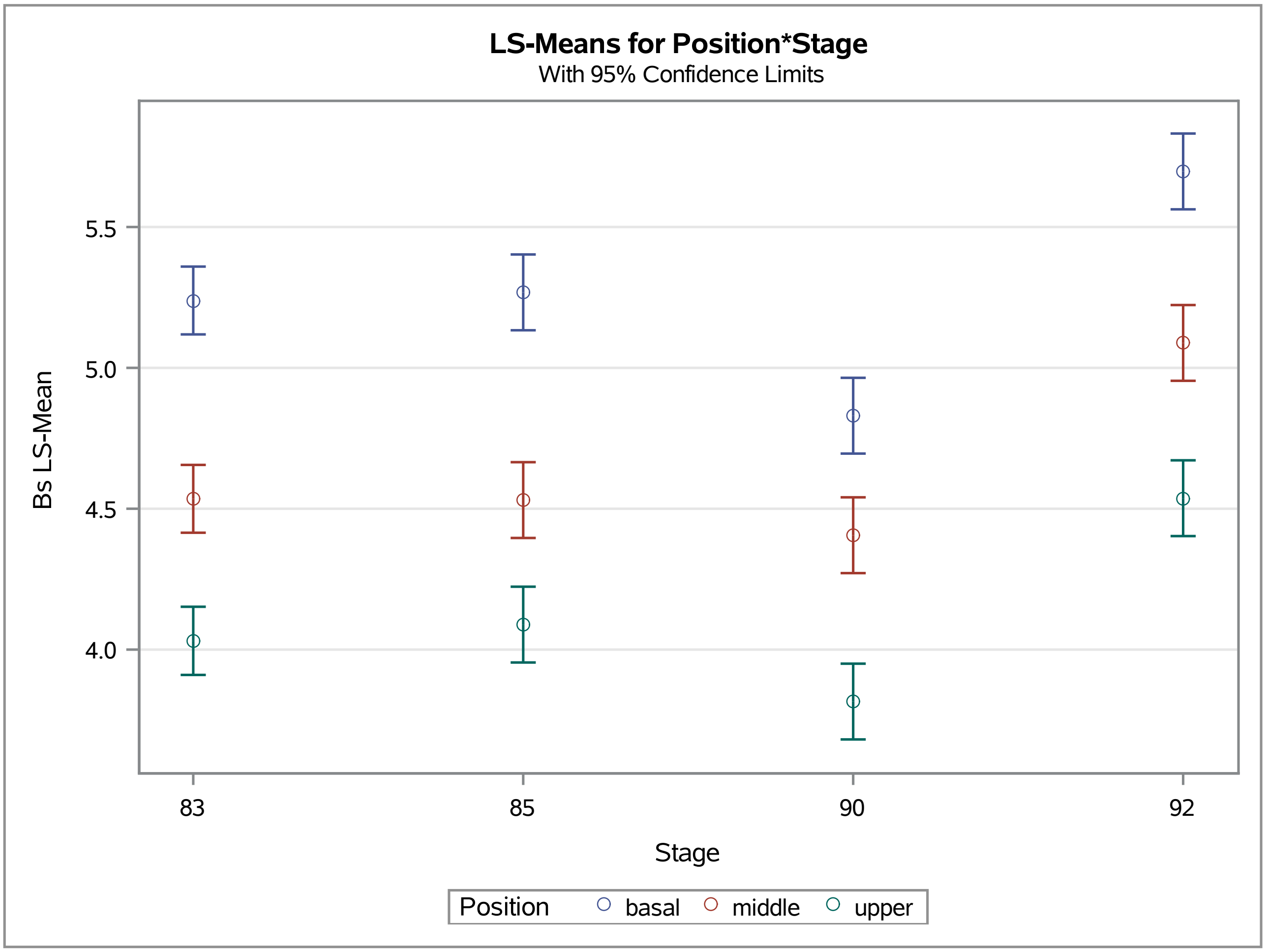

Note 5 (Ketos):
This is the plot of the averages (LS-means) for each cell of the interaction effect 'Position' x 'Stage'. The rank order of Bs values at the three stem segment positions is the same at each BBCH stage.

The GLIMMIX Procedure

Genotype=Ketos

These tests evaluate the effect of Position separately at each Stage. A significant result indicates that LS means differ among positions within that specific stage.

Tests of Effect Slices for Position*Stage Sliced By Stage

Stage	Num DF	Den DF	F Value	Pr > F
83	2	154.1	376.40	<.0001
85	2	154.1	184.63	<.0001
90	2	154.1	135.10	<.0001
92	2	154.1	175.11	<.0001

Note 2 (Ketos):
This is the test of simple effects. The stem segment 'Position' factor has a highly significant effect at each level of the 'Stage' factor.

Simple Effect Comparisons of Position*Stage Least Squares Means By Stage Adjustment for Multiple Comparisons: SMM

Simple Effect Level	Position	Position	Estimate	Standard Error	DF	t Value	Pr > |t|	Adj P	Alpha	Lower	Upper	Adj Lower	Adj Upper
**Stage 83**	basal	middle	0.7040	0.04385	154.1	16.06	<.0001	<.0001	0.05	0.6174	0.7906	0.5982	0.8098
**Stage 83**	basal	upper	1.2081	0.04429	154.2	27.28	<.0001	<.0001	0.05	1.1206	1.2956	1.1013	1.3150
**Stage 83**	middle	upper	0.5041	0.04429	154.2	11.38	<.0001	<.0001	0.05	0.4166	0.5916	0.3972	0.6110
**Stage 85**	basal	middle	0.7373	0.06201	154.1	11.89	<.0001	<.0001	0.05	0.6148	0.8598	0.5877	0.8869
**Stage 85**	basal	upper	1.1793	0.06201	154.1	19.02	<.0001	<.0001	0.05	1.0568	1.3018	1.0297	1.3289
**Stage 85**	middle	upper	0.4420	0.06201	154.1	7.13	<.0001	<.0001	0.05	0.3195	0.5645	0.2924	0.5916
**Stage 90**	basal	middle	0.4243	0.06201	154.1	6.84	<.0001	<.0001	0.05	0.3018	0.5468	0.2747	0.5739
**Stage 90**	basal	upper	1.0147	0.06201	154.1	16.36	<.0001	<.0001	0.05	0.8922	1.1372	0.8651	1.1644
**Stage 90**	middle	upper	0.5904	0.06201	154.1	9.52	<.0001	<.0001	0.05	0.4679	0.7129	0.4408	0.7400
**Stage 92**	basal	middle	0.6087	0.06201	154.1	9.82	<.0001	<.0001	0.05	0.4862	0.7312	0.4591	0.7584
**Stage 92**	basal	upper	1.1600	0.06201	154.1	18.71	<.0001	<.0001	0.05	1.0375	1.2824	1.0103	1.3096
**Stage 92**	middle	upper	0.5512	0.06201	154.1	8.89	<.0001	<.0001	0.05	0.4287	0.6737	0.4016	0.7008

Note 3 (Ketos):
The table above shows the multiple comparisons among the levels of the stem segment 'Position' factor at each level of the 'Stage' factor (that is, within the test of simple effects).

This table reports pairwise comparisons among Positions within each Stage, with multiplicity correction (SMM). Positive estimates indicate that the first Position listed has a higher LS mean than the second. Significance levels are adjusted and correspond to the simple effects tests shown above.

The GLIMMIX Procedure

Genotype=Tibet

Model Information	
**Data Set**	WORK.IMPORT
**Response Variable**	Bs
**Response Distribution**	Lognormal
**Link Function**	Identity
**Variance Function**	Default
**Variance Matrix**	Not blocked
**Estimation Technique**	Restricted Maximum Likelihood
**Degrees of Freedom Method**	Kenward-Roger2
**Fixed Effects SE Adjustment**	Kenward-Roger2

Class Level Information		
**Class**	**Levels**	**Values**
**Genotype**	1	Tibet
**Position**	3	basal middle upper
**Stage**	4	83 85 88 92
**stem**	15	1 2 3 4 5 6 7 8 9 10 11 12 13 14 15

**Number of Observations Read**	180
**Number of Observations Used**	171

Dimensions	
**G-side Cov. Parameters**	1
**R-side Cov. Parameters**	1
**Columns in X**	20
**Columns in Z**	60
**Subjects (Blocks in V)**	1
**Max Obs per Subject**	171

Optimization Information	
**Optimization Technique**	Dual Quasi-Newton
**Parameters in Optimization**	1
**Lower Boundaries**	1
**Upper Boundaries**	0
**Fixed Effects**	Profiled
**Residual Variance**	Profiled
**Starting From**	Data

The GLIMMIX Procedure

Genotype=Tibet

Iteration History					
**Iteration**	**Restarts**	**Evaluations**	**Objective Function**	**Change**	**Max Gradient**
**0**	**0**	4	33.517357318	.	2.538447
**1**	**0**	2	33.229944526	0.28741279	0.982819
**2**	**0**	2	33.143637358	0.08630717	0.230616
**3**	**0**	2	33.139288692	0.00434867	0.029872
**4**	**0**	2	33.139217402	0.00007129	0.000778
**5**	**0**	2	33.139217353	0.00000005	2.544E-6

Convergence criterion (GCONV=1E-8) satisfied.

Fit Statistics	
**-2 Res Log Likelihood**	33.14
**AIC (smaller is better)**	37.14
**AICC (smaller is better)**	37.22
**BIC (smaller is better)**	41.33
**CAIC (smaller is better)**	43.33
**HQIC (smaller is better)**	38.78
**Generalized Chi-Square**	4.80
**Gener. Chi-Square / DF**	0.03

Covariance Parameter Estimates		
**Cov Parm**	**Estimate**	**Standard Error**
**stem(Stage)**	0.06133	0.01382
**Residual**	0.03018	0.004220

Type III Tests of Fixed Effects				
**Effect**	**Num DF**	**Den DF**	**F Value**	**Pr > F**
**Position**	2	103.4	464.14	<.0001
**Stage**	3	55.47	9.72	<.0001
**Position*Stage**	6	103.3	2.69	0.0182

Note 1 (Tibet):
This is the ANOVA table.
The stem segment 'Position' factor has a highly significant effect.

Note 4 (Tibet):
This is the ANOVA table.
The interaction effect 'Position' x 'Stage' is signficant, but not highly significant.

The GLIMMIX Procedure

Genotype=Tibet

Position*Stage Least Squares Means									
Position	Stage	Estimate	Standard Error	DF	t Value	Pr > |t|	Alpha	Lower	Upper
basal	83	4.8811	0.07811	85.93	62.49	<.0001	0.05	4.7258	5.0364
basal	85	4.7121	0.07811	85.93	60.33	<.0001	0.05	4.5568	4.8673
basal	88	4.6008	0.07811	85.93	58.91	<.0001	0.05	4.4455	4.7561
basal	92	5.1705	0.07811	85.93	66.20	<.0001	0.05	5.0153	5.3258
middle	83	4.5268	0.07811	85.93	57.96	<.0001	0.05	4.3716	4.6821
middle	85	4.2802	0.07811	85.93	54.80	<.0001	0.05	4.1249	4.4354
middle	88	4.1298	0.07811	85.93	52.87	<.0001	0.05	3.9745	4.2851
middle	92	4.6014	0.07811	85.93	58.91	<.0001	0.05	4.4462	4.7567
upper	83	3.9643	0.07939	90.2	49.93	<.0001	0.05	3.8065	4.1220
upper	85	3.8070	0.07811	85.93	48.74	<.0001	0.05	3.6517	3.9623
upper	88	3.5108	0.08446	106.5	41.57	<.0001	0.05	3.3434	3.6783
upper	92	3.9537	0.08446	106.5	46.81	<.0001	0.05	3.7862	4.1211

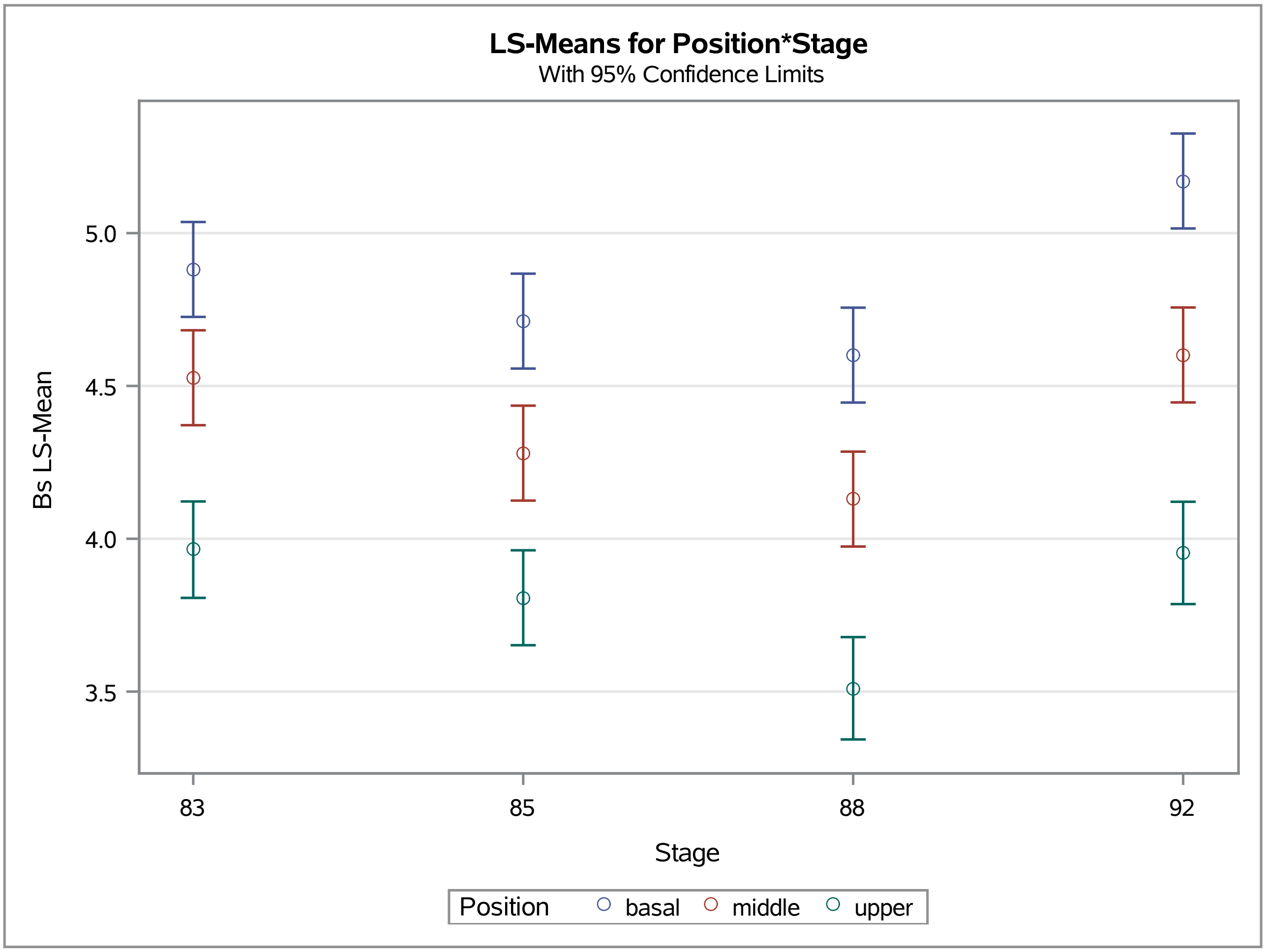

Note 5 (Tibet):
This is the plot of the averages (LS-means) for each cell of the interaction effect 'Position' x 'Stage'. The rank order of Bs values at the three stem segment positions is the same at each BBCH stage.

The GLIMMIX Procedure

Genotype=Tibet

Tests of Effect Slices for Position*Stage Sliced By Stage				
Stage	Num DF	Den DF	F Value	Pr > F
83	2	102.7	100.53	<.0001
85	2	102.3	101.85	<.0001
88	2	104	117.69	<.0001
92	2	104	147.54	<.0001

Note 2 (Tibet):
This is the test of simple effects. The stem segment 'Position' factor has a highly significant effect at each level of the 'Stage' factor.

Simple Effect Comparisons of Position*Stage Least Squares Means By Stage Adjustment for Multiple Comparisons: SMM													
Simple Effect Level	Position	Position	Estimate	Standard Error	DF	t Value	Pr > |t|	Adj P	Alpha	Lower	Upper	Adj Lower	Adj Upper
**Stage 83**	basal	middle	0.3542	0.06344	102.3	5.58	<.0001	<.0001	0.05	0.2284	0.4801	0.2004	0.5081
**Stage 83**	basal	upper	0.9168	0.06501	103	14.10	<.0001	<.0001	0.05	0.7879	1.0458	0.7591	1.0745
**Stage 83**	middle	upper	0.5626	0.06501	103	8.65	<.0001	<.0001	0.05	0.4336	0.6915	0.4049	0.7203
**Stage 85**	basal	middle	0.4319	0.06344	102.3	6.81	<.0001	<.0001	0.05	0.3061	0.5577	0.2780	0.5858
**Stage 85**	basal	upper	0.9051	0.06344	102.3	14.27	<.0001	<.0001	0.05	0.7793	1.0309	0.7512	1.0590
**Stage 85**	middle	upper	0.4732	0.06344	102.3	7.46	<.0001	<.0001	0.05	0.3474	0.5990	0.3193	0.6271
**Stage 88**	basal	middle	0.4710	0.06344	102.3	7.42	<.0001	<.0001	0.05	0.3452	0.5968	0.3171	0.6249
**Stage 88**	basal	upper	1.0900	0.07111	105.1	15.33	<.0001	<.0001	0.05	0.9490	1.2310	0.9175	1.2624
**Stage 88**	middle	upper	0.6190	0.07111	105.1	8.70	<.0001	<.0001	0.05	0.4780	0.7600	0.4465	0.7914
**Stage 92**	basal	middle	0.5691	0.06344	102.3	8.97	<.0001	<.0001	0.05	0.4433	0.6949	0.4152	0.7230
**Stage 92**	basal	upper	1.2169	0.07111	105.1	17.11	<.0001	<.0001	0.05	1.0759	1.3579	1.0444	1.3893
**Stage 92**	middle	upper	0.6478	0.07111	105.1	9.11	<.0001	<.0001	0.05	0.5068	0.7887	0.4753	0.8202

Note 3 (Tibet):
The table above shows the multiple comparisons among the levels of the stem segment 'Position' factor at each level of the 'Stage' factor (that is, within the test of simple effects).
