# Supplementary material for: Barley Stem Bending Resistance Declines During Maturation, Then Peaks in Ripe, Dry Plants
Source: Plants (Basel). 2026 Apr 17;15(8):1234. doi: 10.3390/plants15081234 (PMC13119592; doi:10.3390/plants15081234)
Supplement: Supplementary file 1 [file plants-15-01234-s001.zip › Supplementary Figures.pdf]

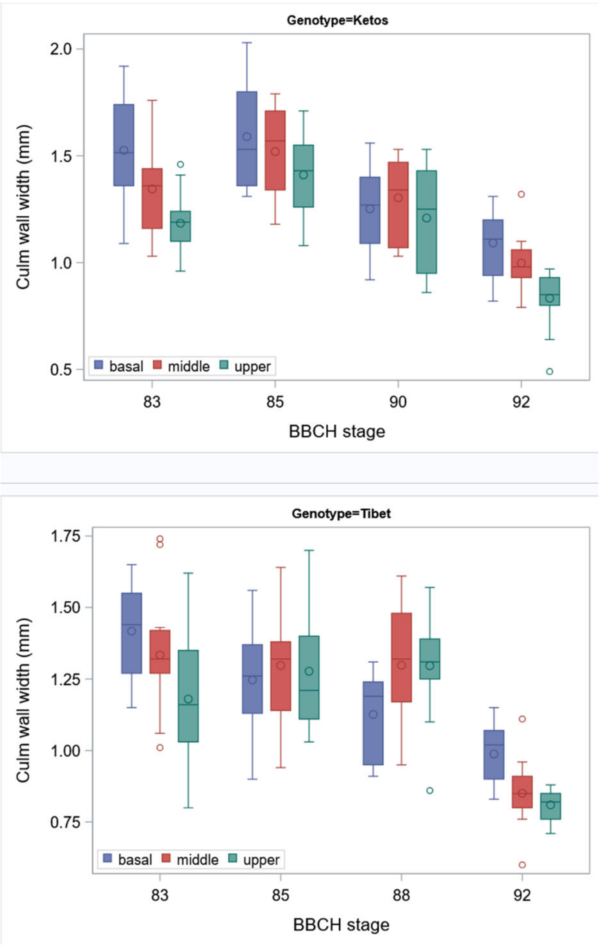

Figure S1. Culm wall thickness ($r_e - r_i$) at pulvinus. Box plots for the basal, middle, and upper stem segments at subsequent phenological development stages (BBCH scale) are shown for cultivar Ketos and landrace Tibet-A4. Measurements were carried out immediately after collecting at all stages but the last, for which it was measured after further drying at RT for over five months. Each box shows the interquartile range (IQR), from the 25th percentile (Q1) to the 75th percentile (Q3). Inside each box, a short line represents the median, and the circle is the mean value. The whiskers extend to the smallest and largest values within 1.5 × IQR of the box. Points beyond the whiskers are plotted as outliers.

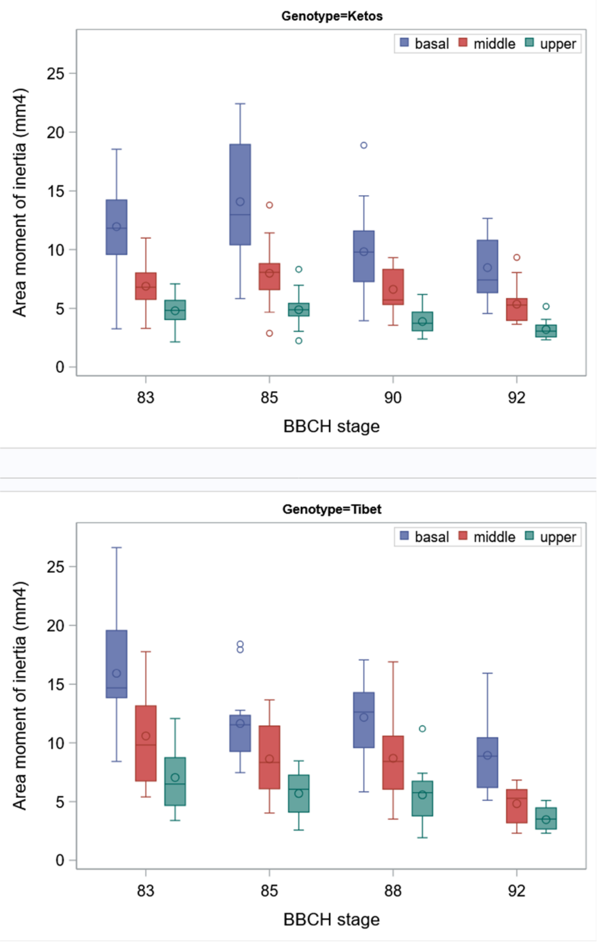

Figure S2. Area moment of inertia (*I*) just below the node. Box plots for the basal, middle, and upper stem segments at subsequent phenological development stages (BBCH scale) are shown for cultivar Ketos and landrace Tibet-A4. Measurements were carried out immediately after collecting at all stages but the last, for which it was measured after further drying at RT for over five months.

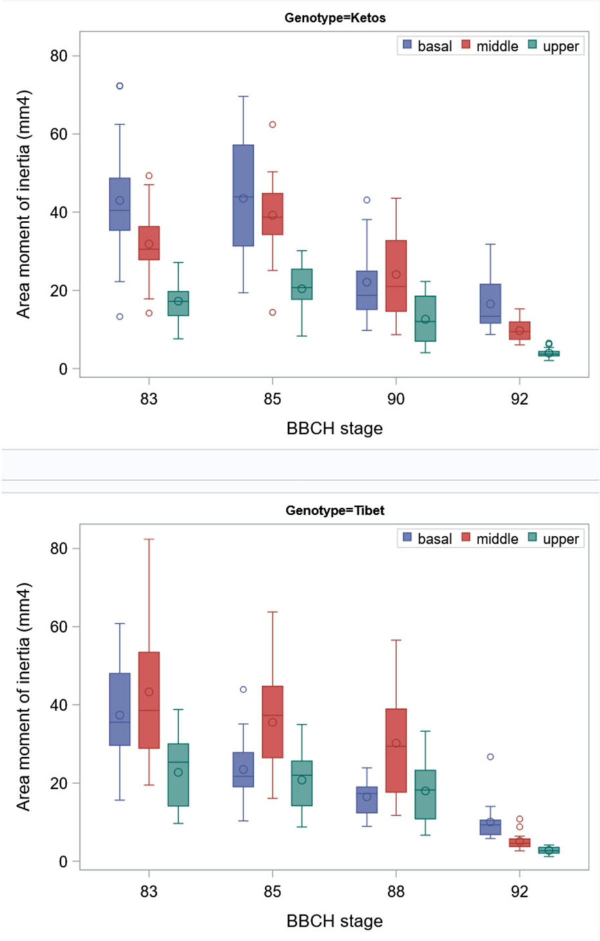

Figure S3. Area moment of inertia (*I*) at pulvinus. Box plots for the basal, middle, and upper stem segments at subsequent phenological development stages (BBCH scale) are shown for cultivar Ketos and landrace Tibet-A4. Measurements were carried out immediately after collecting at all stages but the last, for which it was measured after further drying at RT for over five months.
